# A Methodological Framework for Non-Invasive Body-Composition Phenotyping in Young Adults: Integrating Bioelectrical Impedance and Patient Similarity Networks

**DOI:** 10.3390/life16081336

**Published:** 2026-08-14

**Authors:** Róbert László Nagy, Bence Bombera, Csongor István Szepesi, Nóra Horváth, Viktor Rekenyi, László Róbert Kolozsvári

**Affiliations:** 1 Department of Family and Occupational Medicine, Faculty of Medicine, University of Debrecen, 4032 Debrecen, Hungary; nagy.robert@hook.hu (R.L.N.); bence.bombera@gmail.com (B.B.); szepesi.csongor@med.unideb.hu (C.I.S.); horvath.nora@med.unideb.hu (N.H.); rekenyi.viktor@med.unideb.hu (V.R.); 2 The National Union of Students in Hungary (HÖOK), 1055 Budapest, Hungary

**Keywords:** visceral adiposity, body composition, bioelectrical impedance analysis, cardiometabolic risk, BMI, BIA-defined TOFI-like phenotype, Metabolically Unhealthy Obesity, patient similarity network, unsupervised clustering, young adults

## Abstract

Body mass index (BMI) does not capture fat distribution or muscle–fat heterogeneity, so adverse patterns go undetected. We present a methodological framework—not a validated prediction tool—combining three laboratory-free constructs: an Office-Based Framingham cardiovascular risk score, a modified proxy-based FINDRISC, and a direct segmental multi-frequency bioelectrical impedance analysis (DSM-BIA)-derived Metabolically Unhealthy Obesity (MUO) index, with a weighted patient similarity network. We tested six predefined hypotheses in 1684 young adults (mean age 22.5 ± 8.3 years; 50.2% female). Framingham was applied off-label below 30 years, so its outputs give only relative within-cohort ordering; unavailable FINDRISC items were scored zero, so standard FINDRISC categories do not apply. BMI-defined obesity occurred in 6.9%, high visceral fat in 21.7%, high MUO in 21.8%, a BIA-defined TOFI (thin outside, fat inside)-like phenotype in 5.2%, and a sarcopenic-obesity-like phenotype in 9.9%. Visceral fat correlated with percent body fat (r = 0.855). The network resolved nine interpretable communities (modularity Q = 0.63; permutation *p* = 0.005), including a BIA-defined TOFI-like community (cross-validated AUC = 0.93); k-means, hierarchical, PCA and UMAP clustering recovered convergent axes. All hypotheses were supported, indicating internal construct consistency, not external validation. Laboratory, imaging and longitudinal validation is required before any diagnostic or prognostic claim.

## 1. Introduction

### 1.1. Background

Cardiovascular diseases (CVD) and Type 2 Diabetes Mellitus (T2DM) constitute leading causes of morbidity and mortality worldwide, with their prevalence increasing due to demographic and lifestyle changes [1,2,3]. Early identification of at-risk individuals enables targeted preventive interventions, potentially reducing disease burden and healthcare costs [4]. Cardiovascular risk increasingly accumulates from young adulthood, a stage at which prevention offers the greatest lifetime benefit [5,6], yet conventional screening centred on body mass index and the metabolic syndrome may underestimate risk driven by visceral adiposity [7,8,9,10].

Several validated risk prediction models have been developed over decades of epidemiological research. The Framingham Heart Study, initiated in 1948, produced widely used cardiovascular risk equations validated across multiple populations [11]. Similarly, the Finnish Diabetes Risk Score (FINDRISC) provides a non-invasive tool for T2DM risk assessment validated in over 30 countries [12]. In the present laboratory-free screening setting, we apply a modified proxy-based FINDRISC-like index, which is not a validated implementation of the original instrument but rather an internally consistent approximation adapted to the available data. Because several original FINDRISC components were unavailable and systematically set to zero, this proxy-based index should be interpreted strictly as a relative, internally consistent risk indicator rather than a validated diabetes risk prediction tool. Consequently, its values are not directly comparable to standard FINDRISC thresholds reported in the literature.

In parallel, the International Diabetes Federation (IDF) metabolic syndrome criteria provide a standardised conceptual framework for identifying individuals with clustered metabolic abnormalities [13], further emphasising the importance of multidimensional risk assessment beyond single indicators.

These considerations motivate body-composition-based screening at the population scale, particularly in young adults, among whom early prevention offers the greatest lifetime benefit. Building on this rationale and on our earlier work in this programme—in a previous study of 285 young adults, we showed that combining BIA-derived body composition with BMI uncovers elevated body fat that BMI alone misses [14]—the present study substantially extends that observation to a large nationwide cohort (N = 1684) and integrates non-invasive cardiometabolic risk scoring with patient similarity network stratification.

### 1.2. Rationale for Integration

Despite the availability of these validated tools, their clinical application often occurs in isolation. Modern computational approaches, particularly network-based analytics, offer opportunities to integrate multiple risk dimensions and identify patient subgroups with similar clinical profiles [15,16,17]. Patient similarity networks (PSNs) have emerged as a promising network-based approach for precision medicine research, supporting data-driven patient stratification based on multidimensional similarity patterns [18].

Two features distinguish the present framework from existing bioimpedance-based screening strategies. First, earlier work has used bioimpedance mainly descriptively—reporting that BMI misclassifies adiposity, or that visceral fat tracks with metabolic abnormalities—without embedding the device output in an explicit, falsifiable evaluation design. Here, direct segmental multi-frequency bioelectrical impedance analysis (DSM-BIA) output is combined with two office-based risk instruments in a single laboratory-free pipeline whose behaviour is then tested against six pre-specified quantitative criteria, so that the framework can in principle fail. Second, conventional multivariate approaches such as principal component analysis (PCA), k-means and hierarchical clustering require the number of subgroups to be fixed in advance, and they provide no direct test of whether the resulting partition is stronger than would be expected by chance. A patient similarity network represents each participant as a node in a graph of pairwise multidimensional similarity; the number of subgroups therefore emerges from the graph topology, the strength of the partition is quantified by modularity, and that modularity can be compared with a degree-preserving null model. The graph representation also preserves the continuity between subgroups, which matters when risk is distributed along gradients rather than in discrete classes, as is typical of a young screening population. We did not assume that this representation is superior: the network partition is benchmarked directly against conventional clustering and dimensionality-reduction pipelines in Section 3.7.1.

### 1.3. Objectives

The central aim of this study is to develop and internally evaluate a non-invasive screening-oriented framework for cardiometabolic risk phenotyping, and to evaluate whether the framework behaves consistently with its predefined design by testing a set of predefined hypotheses in a predominantly young-adult screening cohort. The framework integrates three components into a single pipeline: (i) non-invasive risk scoring (Office-Based Framingham 10-year cardiovascular disease risk, a modified proxy-based FINDRISC and a BIA-derived Metabolically Unhealthy Obesity score); (ii) InBody 270 DSM-BIA body-composition phenotyping; and (iii) a weighted patient similarity network with Louvain community detection. Specifically, we sought to:

Define the framework’s input representation by characterising the distribution of InBody 270 DSM-BIA-derived body-composition parameters and the three integrated non-invasive cardiometabolic risk constructs;

Quantify the extent to which visceral adiposity and metabolic risk are decoupled from body mass index in this cohort, including the prevalence of the BIA-defined TOFI-like (thin outside, fat inside) phenotype as operationally defined by bioimpedance;

Show that the framework’s patient similarity network with Louvain community detection recovers phenotypically interpretable, statistically robust groups;

Assess whether the three integrated risk constructs contribute complementary, non-redundant information to the framework.

The integrated framework—comprising its risk-scoring, body-composition phenotyping and network-stratification logic—is the object under evaluation in this study; the biological phenotypes it surfaces constitute the evidence used to assess whether the framework performs as intended. To make this demonstration explicit rather than descriptive, we predefined the hypotheses defined in Section 1.4 and evaluated them in Section 3.9.

### 1.4. Hypotheses

To evaluate whether the framework behaves consistently with its predefined design rather than merely to describe the cohort, we predefined six hypotheses, each tied to a concrete, falsifiable prediction about the framework’s behaviour and to an explicit confirmation criterion that is evaluated in Section 3.9. A hypothesis was considered supported only if its predefined quantitative criterion was met.

**H1**  (Hidden-risk detection)**.** *The framework identifies cardiometabolic risk phenotypes, BIA-defined TOFI-like, sarcopenic-obesity-like and high Metabolically Unhealthy Obesity (MUO) burden, in participants who are classified as low-risk by BMI. Confirmation criterion: a clinically non-trivial prevalence (≥5%) of at least one hidden-risk phenotype within the normal-weight stratum.*

**H2**  (Construct consistency/dose–response)**.** *Framework-derived risk scores increase monotonically along established risk gradients. Confirmation criterion: monotonically rising mean MUO score and visceral fat level across BMI categories and across age groups (one-way ANOVA p < 0.05).*

**H3**  (Complementarity/non-redundancy)**.** *The three integrated risk constructs capture distinct, non-redundant dimensions of risk. Confirmation criterion: low pairwise agreement at elevated-risk thresholds (Cohen’s κ ≈ 0; Lin’s concordance correlation coefficient ≤ 0.30).*

**H4**  (Discrimination)**.** *The framework’s multivariable feature model discriminates the hidden-risk (BIA-defined TOFI-like) phenotype. Confirmation criterion: out-of-fold area under the ROC curve ≥ 0.85 from stratified five-fold cross-validation with an acceptable model fit (McFadden pseudo-R^2^ ≥ 0.30).*

**H5**  (Network stratification validity)**.** *The patient similarity network recovers statistically robust, reproducible and phenotypically interpretable groups. Confirmation criterion: modularity Q > 0.3 that significantly exceeds a degree-preserving configuration-model null (permutation p < 0.05), with a deterministic, seed-fixed partition.*

**H6**  (Sex-calibration invariance)**.** *The composite framework risk burden is calibrated to be sex-invariant despite physiological sex differences in fat distribution. Confirmation criterion: no significant sex difference in the composite MUO score (p > 0.05, negligible effect size) despite large sex differences in its constituent body-composition inputs.*

## 2. Materials and Methods

### 2.1. Study Design and Data Source

**Figure 1:**
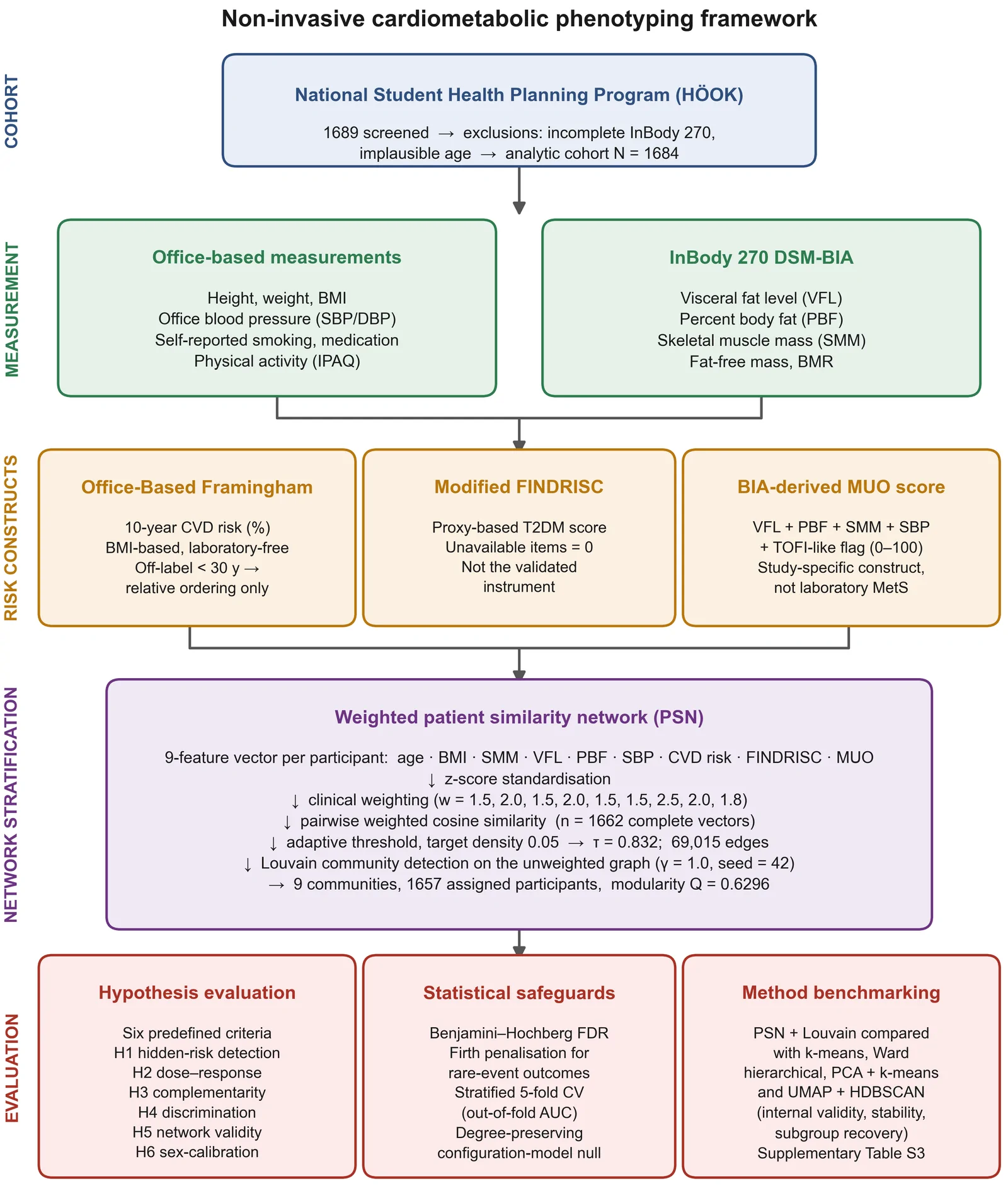
Schematic overview of the analytical workflow. Office-based measurements and InBody 270 DSM-BIA (InBody Co., Ltd., Seoul, Republic of Korea) parameters feed three non-invasive risk constructs (Office-Based Framingham 10-year cardiovascular disease risk, modified proxy-based FINDRISC, and the BIA-derived MUO score). These, together with the body-composition variables, form the nine-feature vector used to build the weighted patient similarity network. Louvain community detection on the thresholded network yields the phenotypic communities, which are then evaluated against six predefined hypotheses, with the statistical safeguards and the benchmarking against conventional clustering approaches shown in the lower row. DSM-BIA, direct segmental multi-frequency bioelectrical impedance analysis; BMR, basal metabolic rate; MUO, Metabolically Unhealthy Obesity; TOFI, thin outside fat inside; VFL, visceral fat level; PBF, percent body fat; SMM, skeletal muscle mass; SBP, systolic blood pressure; DBP, diastolic blood pressure; MetS, metabolic syndrome; FDR, false discovery rate; CV, cross-validation.Solid arrows indicate the downward flow of the data processing and analysis steps.

This cross-sectional, observational study analysed data from 1684 participants with complete screening data attending the National Student Health Planning Programme. Data were collected between January 2025 and February 2026 as part of this nationwide initiative. The programme is coordinated by the National Union of Students in Hungary (HÖOK), an organisation dedicated to fostering a comprehensive healthy lifestyle, including the physical and mental well-being of young adults across the country. HÖOK provided the nationwide logistical infrastructure and the participant recruitment network across higher education institutions. The scientific protocol, the clinical data processing and the statistical analyses were conducted independently by the clinical research team. For each participant, we recorded anthropometric measurements, office blood pressure, self-reported physical activity (International Physical Activity Questionnaire, IPAQ) [19], and InBody 270 DSM-BIA (InBody Co., Ltd., Seoul, Republic of Korea) body-composition parameters [20]. Body mass index was taken from the device output, or computed from height and weight when not directly available. Data were processed and analysed with a custom, open-source pipeline implemented in Python version 3.12 (pandas, NumPy, SciPy, scikit-learn, NetworkX, statsmodels) [21]. Figure 1 summarises the complete analytical workflow, from cohort assembly through risk-construct derivation to network stratification and hypothesis evaluation.

### 2.2. Risk Calculation Models

#### 2.2.1. Office-Based Framingham CVD Risk Score

The 10-year cardiovascular disease risk is calculated using the BMI-based (Office-Based) Framingham equation from D’Agostino et al. (2008) [11]. This version is specifically validated for primary care settings where laboratory lipid profiles are unavailable:

In this young cohort (mean age 22.5 years), application of the Framingham equation is exploratory and off-label for participants under 30 years of age, because the original Office-Based Framingham model was derived and validated in adults aged 30–74 years [11]. Accordingly, the outputs of this equation are used throughout the present manuscript solely as a relative, within-cohort ordering of cardiovascular risk. The category labels used in the Results denote strata of that ordering and must not be read as calibrated 10-year probabilities for individual participants. In this laboratory-free cohort, the smoking and diabetes terms of the equation were derived from self-reported, physician-diagnosed status; participants without a recorded diagnosis were coded as non-smoking or non-diabetic (0), respectively, a conservative assumption consistent with the handling of the other unavailable covariates.

Model Specification: 

For males:

$$L\;=\;3.11296\;\cdot ln\left(age\right)+\;0.79277\;\cdot ln\left(BMI\right)+\;\beta_{SBP}\cdot ln\left(SBP\right)+\;0.70953\cdot smoking\;+\;0.53160\;\cdot diabetes$$

For females:

$$L\;=\;2.72107\;\cdot ln\left(age\right)+\;0.51125\;\cdot ln\left(BMI\right)+\;\beta_{SBP}\cdot ln\left(SBP\right)+\;0.61868\cdot smoking\;+\;0.77763\;\cdot diabetes$$

where $\beta_{SBP}\;$ differs based on treatment status:

Males: treated = 1.92672, untreated = 1.85508,

Females: treated = 2.88267, untreated = 2.81291.

Risk Calculation:

$$P_{10yr}=1-S_{0}^{exp(L-\bar{L})}$$

where:

$S_{0}$ = baseline 10-year survival (0.88431 males, 0.94833 females),

$\bar{L}$ = mean coefficient sum (23.9388 males, 26.0145 females).

Risk strata follow the 2021 European Society of Cardiology (ESC) guidelines on cardiovascular disease prevention in clinical practice [22] and are reproduced in Supplementary Table S1.

Validation: These metrics refer to the original Office-Based Framingham model: C-statistic 0.763 (males), 0.793 (females); original derivation cohort n = 8491, ages 30–74 years.

#### 2.2.2. Modified FINDRISC (Proxy-Based Finnish Diabetes Risk Score)

A modified, proxy-based FINDRISC-like score was calculated for relative, within-cohort ordering of Type 2 Diabetes risk, adapted from the original Finnish Diabetes Risk Score [12]. This is not a validated implementation of the FINDRISC and does not provide a calibrated absolute 10-year diabetes risk estimate. Because this laboratory-free screening dataset lacks blood glucose history and systematically recorded family history, these items are conservatively scored as zero or omitted (detailed below); the resulting instrument is referred to throughout as the modified FINDRISC:

The scoring components of the modified instrument are listed in Supplementary Table S2.

Total Score Range: 0–26 points.

For reference only, the risk categories and approximate 10-year diabetes risks of the original, validated FINDRISC are reproduced in Supplementary Table S3. These categories are calibrated for the complete instrument and are therefore not applicable to the modified proxy-based score used here; they are not used to interpret any result in this study.

Waist Estimation (when unavailable)**:** If waist circumference is not measured, the framework estimates it using the empirical relationship:

$${\mathrm{Waist}}_{est}=2.5\cdot \mathrm{BMI}+40\;(\mathrm{cm})$$

Proxy Variables for Missing FINDRISC Components:

Because laboratory glucose values are unavailable in this non-invasive screening setting, the proxy estimation strategies summarised in Supplementary Table S4 were employed. Antihypertensive treatment was proxied by self-reported, physician-diagnosed hypertension or reported antihypertensive medication, which was present in 2.6% (44/1684) of participants, consistent with treatment rates reported for this age group [23,24].

Family History: First-degree family history of T2DM and hypertension was not systematically recorded in this screening programme and was therefore scored conservatively as 0 for all participants. This introduces a downward bias in the FINDRISC, consistent with the screening tool’s conservative design philosophy of minimising false positives [25].

Scoring Adjustment: Components with unavailable data receive 0 points (conservative approach), resulting in potential underestimation of true FINDRISC. This conservative bias is clinically acceptable for a screening tool as it minimises false positives while still flagging participants with the highest proxy scores.

Validation: These figures refer to the original, validated FINDRISC: sensitivity 78%, specificity 77% at a score ≥11, and AUC 0.72–0.87 depending on population. They do not describe the proxy-based score used in the present study, which has not been externally validated.

#### 2.2.3. MUO (Metabolically Unhealthy Obesity) Risk Assessment

Since laboratory values (triglycerides, HDL, fasting glucose) are unavailable in this non-invasive screening setting, we replace traditional Metabolic Syndrome (MetS) criteria with an operational, BIA-derived Metabolically Unhealthy Obesity (MUO) risk score based on InBody 270 DSM-BIA body composition parameters. This BIA-derived MUO score is a study-specific construct and is not equivalent to laboratory-defined metabolic syndrome or clinically confirmed Metabolically Unhealthy Obesity:

MUO Risk Components (InBody 270-derived):
**Component****Source****Threshold****Score Contribution**Visceral Fat Level (VFL)InBody 270≥10 (high)/7–10 (moderate)40/20 pointsPercent Body Fat (PBF)InBody 270M: ≥25% (high)/20–25% (mod); F: ≥33% (high)/25–33% (mod)20/10 pointsSkeletal Muscle Mass (SMM)InBody 270PBF ≥ sex-specific obesity threshold AND SMM < 32 kg(M)/20 kg(F)15 points (sarcopenic)Systolic Blood PressureOffice measurement≥130 mmHg15 pointsBIA-Defined TOFI-Like PhenotypeCalculatedBMI < 25 AND VFL ≥ 1015 points (hidden risk)

Sex-Specific Percent Body Fat (PBF) Thresholds: PBF thresholds are differentiated by sex to reflect established physiological differences in body composition. Male obesity is defined at PBF ≥ 25% and female obesity at PBF ≥ 33%, consistent with ACE (American Council on Exercise) body fat norms and Gallagher et al. (2000) [26]. Two distinct operational rules involving skeletal muscle mass (SMM) are used, and they are reported separately here to avoid ambiguity. (i) Within the MUO score, a sarcopenic component contributes 15 points when PBF exceeds the sex-specific obesity threshold AND SMM falls below 32 kg (males) or 20 kg (females), each approximately one standard deviation below the corresponding sex-specific cohort mean. (ii) The standalone prevalence of a sarcopenic-obesity-like phenotype uses a percent-body-fat-anchored rule: PBF ≥ 30% together with SMM below 30 kg (males) or 23 kg (females). Rule (ii) identifies 166 participants (9.9%); rule (i) would identify 91 (5.4%). Both are operational, cohort-anchored approximations. They are informed by, but not equivalent to, the consensus criteria of the revised European Working Group on Sarcopenia in Older People (EWGSOP2; Cruz-Jentoft et al. 2019) and by the ESPEN/EASO consensus on sarcopenic obesity [27,28], both of which require height- or weight-adjusted muscle mass and an objective measure of muscle function; neither the adjustment nor functional testing was available in this screening programme. The phenotype is therefore designated as sarcopenic-obesity-like throughout.

**BIA-defined TOFI-like (Thin Outside, Fat Inside) Detection:**

A key feature is the identification of a BIA-defined TOFI-like phenotype—individuals with normal BMI (<25 kg/m^2^) but elevated visceral fat (VFL ≥ 10). These individuals are not identified by conventional BMI-based screening but carry a body-composition pattern associated with elevated metabolic risk. The VFL ≥ 10 threshold was selected because, according to the device specifications [20], it corresponds to an estimated visceral fat area of ≥100 cm^2^, a well-established clinical cut-off for increased cardiometabolic risk in imaging and impedance studies.

Because visceral fat is assessed by bioelectrical impedance rather than by computed tomography (CT) or magnetic resonance imaging (MRI), the phenotype is termed BIA-defined TOFI-like throughout this manuscript.

**MUO Score Calculation:**

$$MUO_{score}=\sum_{i}{\mathrm{component}}_{i}$$

**Risk Categories:**

**MUO Score**

**Category**

**Interpretation**
<30Low RiskRoutine follow-up30–60Moderate RiskCandidate for lifestyle counselling≥60High RiskRisk-enriched phenotype, potential target for early intervention


### 2.3. Patient Similarity Network (PSN) Construction

#### 2.3.1. Feature Vector Representation

Each participant is represented as a d-dimensional feature vector:

$$x_{p}=[x_{1},x_{2},...,x_{d}]$$

**Feature Set (InBody 270-optimised):**

Visceral Fat Level (VFL)—InBody 270,

Systolic Blood Pressure (mmHg),

Skeletal Muscle Mass (SMM)—InBody 270,

Percent Body Fat (PBF)—InBody 270,

Age (years),

BMI (kg/m^2^),

CVD risk (%, Office-Based Framingham output),

T2DM risk (%, FINDRISC output),

MUO score (0–100).

#### 2.3.2. Z-Score Normalisation

All features are standardised to zero mean and unit variance:

$$z_{i}=\frac{x_{i}-\mu_{i}}{\sigma_{i}}$$

This ensures comparability across features with different scales.

#### 2.3.3. Clinical Weighting Schema

Features receive differential weights based on epidemiological reasoning and on InBody 270 measurement precision. Weights are listed below in the order in which the features enter the feature vector defined in Section 2.3.1.
**Feature (Vector Order)****Weight****Rationale**Age (years)1.5Non-modifiable cardiometabolic risk multiplierBMI (kg/m^2^)2.0Conventional anthropometric reference axisSkeletal muscle mass (SMM)1.5Determinant of insulin sensitivity and T2DM riskVisceral fat level (VFL)2.0Strongest metabolic risk marker available from DSM-BIA [29]Percent body fat (PBF)1.5More informative than BMI for adipositySystolic blood pressure (mmHg)1.5Office-Based Framingham core component [11]CVD risk (%, Office-Based Framingham output)2.5Integrated cardiovascular constructT2DM risk (modified FINDRISC output)2.0Integrated diabetes constructMUO score (0–100)1.8Composite body-composition indicator

Rationale: the weights were specified a priori on clinical grounds, with the greatest emphasis on the integrated cardiovascular construct and on the InBody 270 DSM-BIA parameters (VFL, BMI, SMM, PBF) that provide objective, non-invasive metabolic phenotyping without laboratory values. Because these weights are analyst-specified rather than data-derived, the resulting partition is reported alongside unweighted conventional clustering benchmarks (Section 3.7.1), and the network is not presented as an optimal representation of the feature space.

#### 2.3.4. Weighted Cosine Similarity

Pairwise participant similarity is computed as:

$$sim(A,B)=\frac{\sum_{i=1}^{d}w_{i}\cdot a_{i}\cdot b_{i}}{∥w⊙A∥\cdot ∥w⊙B∥}$$

where:

w = weight vector,

⊙ = element-wise multiplication,

∥⋅∥ = Euclidean norm.

Weighted Euclidean and Gaussian (radial basis function) similarity kernels are also implemented in the pipeline and are specified in Supplementary Methods S1; every result reported here uses weighted cosine similarity.

#### 2.3.5. Adaptive Threshold Selection

Rather than using an arbitrary fixed threshold, the framework employs data-driven threshold selection based on adaptive target network density. The target density scales inversely with population size to maintain interpretable average degree (~75 neighbours) across varying sample sizes:

$$\rho_{target}(n)=max\left(0.02,\;min\left(0.15,\;\frac{75}{n}\right)\right)$$

where n is the number of participants. This yields:

$n=100\to \rho_{target}=0.15$ (dense, small population),

$n=500\to \rho_{target}=0.15$ (capped at upper bound),

$n=2000\to \rho_{target}\approx 0.0375$ (sparse, large population),

$n=5000\to \rho_{target}=0.02$ (floor, very large population).

The similarity threshold τ is then derived as:

$$\tau =\mathrm{percentile}\left(S_{upper},(1-\rho_{target})\times 100\right)$$

where $S_{upper}$ is the upper triangle of the pairwise similarity matrix. For the present cohort (n = 1662 participants with a complete feature vector), the adaptive formula yields ρ_target ≈ 0.045; the realised network applied a target density of 0.05, corresponding to an average node degree of ≈83.

Rationale: an excessively dense graph renders all participants mutually similar and is uninformative, whereas an excessively sparse graph fragments into isolated nodes with no community structure. The adaptive range keeps the number of edges manageable while retaining interpretable communities; the threshold is additionally constrained to the interval [0.5, 0.95].

#### 2.3.6. Edge Creation

Edges are created between participants with similarity exceeding threshold:

$$E=\{(p_{i},p_{j}):sim(p_{i},p_{j})\geq \tau ,\;i<j\}$$

Edge weights equal similarity values.

### 2.4. Community Detection

Participant clusters are identified using the Louvain algorithm for modularity optimisation [30,31], implemented via nx.algorithms.community.louvain_communities with deterministic seed (seed = 42) and resolution parameter γ=1.0. Community detection is performed on the unweighted (binary) adjacency of the thresholded similarity graph: an edge encodes a supra-threshold similarity, so the partition is defined by the topology of these links rather than by their continuous weights. This also keeps the observed partition methodologically consistent with the degree-preserving permutation null (below), which is likewise unweighted.

Modularity Q was computed in its standard form on the binary adjacency matrix with resolution parameter γ = 1.0 [30,31]; the full expression and the definition of every symbol are given in Supplementary Methods S1.

Statistical significance of community structure: The observed modularity is compared against a degree-preserving configuration-model null distribution generated by repeated double-edge-swap randomisation of the observed graph (preserving the exact degree sequence). For each null replicate, Louvain community detection is re-run and its modularity recorded; the empirical one-sided *p*-value is computed as $\left(1+\#\{Q_{null}\geq Q_{obs}\})/(k+1\right)$ over k replicates.

Post-processing:

Isolated-vertex exclusion: Vertices with no supra-threshold similarity to any other participant (degree 0) are not assignable to a community and are excluded from the partition.

Sorting: Communities are sorted by size (largest first).

Modularity score (Q): Computed and reported to quantify community structure quality (Q>0.3 indicates well-defined communities).

Fallback: If Louvain is unavailable (older NetworkX versions), label propagation community detection is used as a fallback with the same minimum size filter.

Complexity: O(nlogn), suitable for large participant populations (n>1000).

Community Summary: Each community is characterised by mean values for age, BMI, CVD%, T2DM%, MUO score, and visceral fat level (VFL), enabling phenotype labelling.

Interpretation of Network Communities: Communities represent phenotypically interpretable, data-driven participant subgroups with related risk profiles, potentially indicating:

Metabolically healthy cluster—reassurance, maintain lifestyle.

Lean low-risk clusters—routine follow-up.

Elevated visceral-fat/high-MUO clusters—early prevention target.

Older high-CVD cluster—intensive intervention candidates.

### 2.5. Statistical Analysis

Continuous variables are summarised as mean (standard deviation) or median (interquartile range) and compared between sexes using Welch’s *t*-test with Cohen’s d effect sizes; categorical variables are summarised as counts and percentages. Bivariate associations are quantified with Pearson and Spearman correlation coefficients, with Benjamini–Hochberg false discovery rate (FDR) correction for multiple comparisons. Differences across age groups are assessed by one-way analysis of variance (ANOVA) with eta-squared effect sizes. Associations with binary outcomes are explored using logistic regression, with Firth’s penalised likelihood for rare-event outcomes; models with fewer than approximately ten events per variable [32] are reported as exploratory, descriptive associations only; the discrimination of the multivariable BIA-defined TOFI-like model (Model C) was quantified as the out-of-fold area under the ROC curve from stratified five-fold cross-validation, reported alongside the in-sample value to gauge optimism, in line with current prediction-model guidance [33,34]. Community structure in the patient similarity network is assessed by modularity, with a degree-preserving configuration-model permutation test (k = 200 replicates). A two-sided *p* < 0.05 (FDR-adjusted where applicable) is considered statistically significant. Analyses were performed in Python 3.12. The clustering benchmark reported in Section 3.7.1 additionally used scikit-learn 1.8.0, networkx 3.6.1 and umap-learn 0.5.12; the benchmark script is provided with the submission.

### 2.6. Structural Dependency Between Derived Variables

Several derived variables used in the present framework are not independent but share underlying components. Specifically, the MUO score incorporates visceral fat level, percent body fat, and systolic blood pressure, while these same variables are also included individually in correlation analyses and in the construction of the patient similarity network.

As a result, associations involving these variables partly reflect internal structural consistency rather than independent biological relationships. Correlations and clustering patterns should therefore be interpreted in the light of this shared feature composition, and not as evidence of external validation. To keep this distinction explicit, we use the term construct consistency throughout the Results and Discussion whenever an association is at least partly determined by shared inputs, and we reserve the term validation for comparisons against independent, externally measured criteria—none of which were available in this study.

## 3. Results

### 3.1. Study Population

A total of 1689 participants were screened. After excluding participants with incomplete InBody 270 measurements and implausible age values (retaining 14 ≤ age ≤ 90 with complete BMI and visceral fat data), 1684 participants were included in the final analytic cohort. While the screening programme primarily targeted university students (establishing a predominantly young-adult cohort with a median age of 20 years), the initiative remained open to university staff. The inclusion of this older minority (extending the age range to 71 years) does not confound the primary focus on young adults; rather, it serves as an in-dataset positive control for the patient similarity network, which successfully and autonomously isolated these older participants with elevated risk indicators into a distinct community (Cluster C6), preventing them from skewing the young adult phenotypes. The relatively high proportion of self-reported current smokers (36.0%; Table 1) is consistent with smoking rates reported for young adults in this Central/Eastern European setting and directly increases the computed Office-Based Framingham risk; it should therefore be interpreted in light of the self-report caveat noted in the Limitations (Section 4.6).

**Table 1:** Baseline characteristics of study participants (N = 1684).

Characteristic	Total (N = 1684)	Male (n = 839)	Female (n = 845)	*p*-Value
Demographics				
Age, years, mean (SD)	22.5 (8.3)	23.0 (8.0)	22.1 (8.5)	-
Age, median (IQR)	20 (18–23)	—	—	-
Age range	15–71	—	—	-
Female, n (%)	845 (50.2%)	—	—	-
Anthropometrics				
BMI, kg/m^2^, mean (SD)	23.5 (4.2)	24.8 (4.1)	22.2 (3.8)	<0.001
Normal weight (BMI < 25), n (%)	1187 (70.5%)	—	—	-
Overweight (BMI 25–30), n (%)	380 (22.6%)	—	—	-
Obese (BMI ≥ 30), n (%)	117 (6.9%)	—	—	-
InBody 270 DSM-BIA Parameters				
Visceral Fat Level, mean (SD)	7.0 (4.4)	6.4 (4.4)	7.6 (4.2)	<0.001
VFL < 7 (normal), n (%)	979 (58.1%)	—	—	-
VFL 7–9 (moderate), n (%)	340 (20.2%)	—	—	-
VFL ≥ 10 (high), n (%)	365 (21.7%)	—	—	-
Percent Body Fat (%), mean (SD)	24.1 (9.4)	19.2 (8.0)	28.9 (8.0)	<0.001
Skeletal Muscle Mass (kg), mean (SD)	30.2 (8.2)	36.8 (5.8)	23.7 (3.6)	<0.001
Blood Pressure				
Systolic BP, mmHg, mean (SD)	124.0 (15.7)	132.5 (13.7)	115.5 (12.6)	<0.001
Diastolic BP, mmHg, mean (SD)	77.0 (9.2)	78.2 (9.4)	75.9 (8.8)	<0.001
ESC 2024 hypertension range (≥140/90 office) [35], n (%)	328 (19.7%)	—	—	-
Clinical History (Self-Reported)				
Antihypertensive medication or self-reported HT, n (%)	44 (2.6%)	—	—	-
Family history (T2DM/hypertension)	Not reported	—	—	-
Lifestyle Factors				
Current smoker, n (%)	607 (36.0%)	—	—	-

*p*-values from Welch *t*-test for continuous variables (male vs. female). SD = standard deviation; BMI = Body Mass Index; VFL = visceral fat level; BP = blood pressure; T2DM = Type 2 Diabetes Mellitus; HT = hypertension. Family history of T2DM and hypertension was not systematically recorded in this screening dataset. Cardiovascular risk categories follow the 2021 ESC guidelines on cardiovascular disease prevention in clinical practice [22], whereas the office blood-pressure threshold for hypertension (≥140/90 mmHg) follows the 2024 ESC guidelines for the management of elevated blood pressure and hypertension [35].

### 3.2. Risk Score Distributions

**Figure 2:**
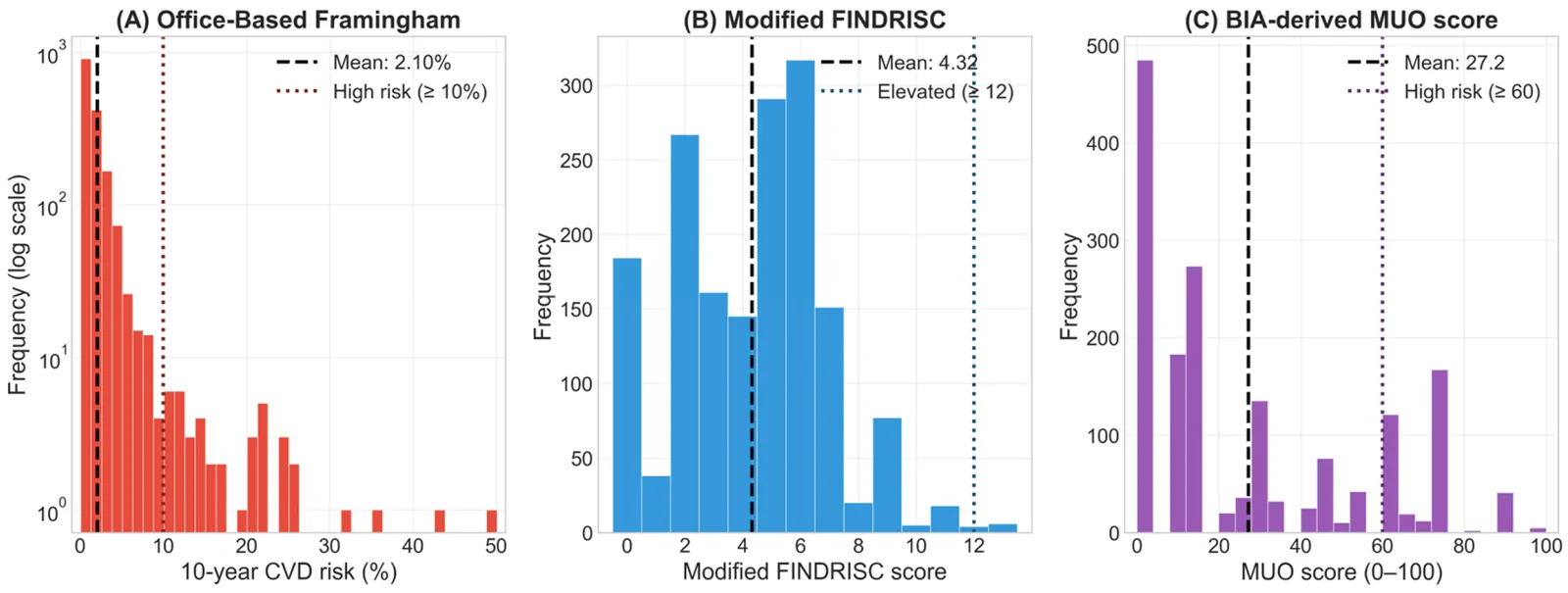
Distribution of the three non-invasive risk constructs across the cohort (N = 1684): (**A**) Office-Based Framingham 10-year cardiovascular disease risk, (**B**) modified FINDRISC, and (**C**) the bioelectrical impedance-derived Metabolically Unhealthy Obesity (MUO) score.

**Figure 3:**
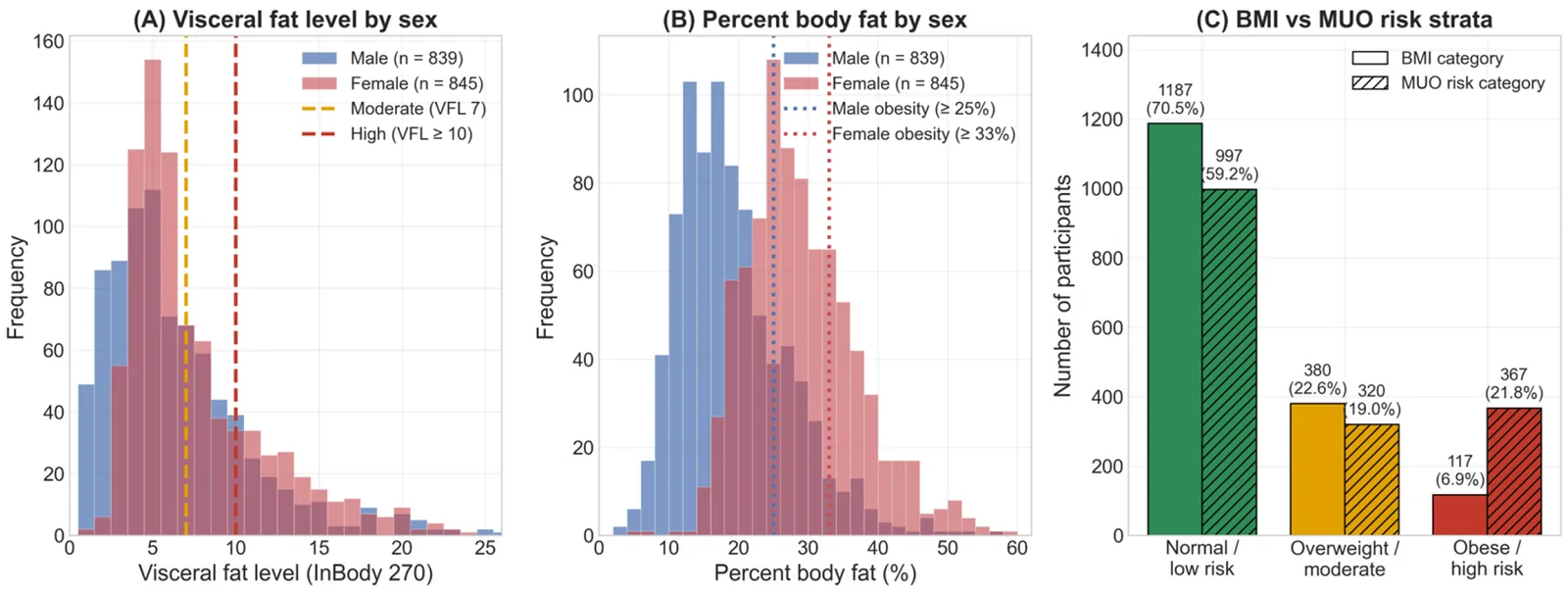
Body-composition profile of the study population (n = 839 male, n = 845 female). (**A**) Visceral fat level by sex, with the moderate (VFL 7) and high (VFL ≥ 10) thresholds. (**B**) Percent body fat by sex, with the sex-specific obesity thresholds (male ≥ 25%, female ≥ 33%). (**C**) Distribution of participants across the three BMI categories (solid bars) and the three MUO risk strata (hatched bars); counts and percentages of the full cohort (N = 1684) are annotated. The two classifications are not equivalent and are deliberately shown side by side: BMI assigns 6.9% of the cohort to its highest category, whereas the body-composition score assigns 21.8%, which is the discrepancy this study addresses. VFL, visceral fat level; MUO, Metabolically Unhealthy Obesity.

The distributions of the three calculated risk constructs across the study population—Office-Based Framingham cardiovascular risk, the modified FINDRISC and the MUO risk score—are shown in Figure 2. The cohort’s body-composition profile, comprising the sex-stratified distributions of visceral fat level and percent body fat together with the BMI and MUO risk strata, is summarised in Figure 3.

#### 3.2.1. Office-Based Framingham CVD Risk

Because of the young mean age of the participants (22.5 years; median 20), the calculated absolute risks were expectedly low. Consistent with the off-label application of the equation below 30 years of age (Section 2.2.1), these values are used exclusively for relative, within-cohort ordering; the category labels in Table 2 denote strata of that ordering rather than calibrated individual 10-year prognoses.

**Table 2:** Distribution of the Office-Based Framingham 10-year cardiovascular disease risk ordering (n = 1662). Category labels denote strata of a relative within-cohort ordering and not calibrated absolute 10-year probabilities (Section 2.2.1).

Risk Category	Threshold	n (%)
Low	<5%	1559 (93.8%)
Moderate	5–10%	62 (3.7%)
High	10–20%	24 (1.4%)
Very High	≥20%	17 (1.0%)
**Summary Statistics**		
Mean (SD)		2.10% (3.34)
Median (IQR)		1.20% (0.70–2.20)

The low overall risk reflects the young age of the participants. Nonetheless, 103 participants (6.2%) fell into the moderate-to-very-high strata of this relative ordering (Table 2), predominantly older males, which highlights sex and age differences in early cardiovascular risk. Blood pressure was available for 1662 participants, which defines the denominator for the Framingham calculation.

#### 3.2.2. Modified FINDRISC T2DM Risk

Reflecting the young age of the cohort and the conservative scoring (family history and glucose history unavailable), only 0.6% (n = 10) of participants reached the highest band of the modified FINDRISC (≥12; Table 3). Because several components of the original instrument were systematically scored as zero, this proportion is not an estimate of diabetes risk prevalence and is not comparable with published FINDRISC prevalences. It identifies, within this cohort only, a small subgroup that would be prioritised for lifestyle counselling if the ordering were confirmed against laboratory measures.

**Table 3:** Distribution of the modified, proxy-based FINDRISC (N = 1684). The score-band labels are retained for descriptive comparability only. The approximate 10-year diabetes risks attached to these bands in the original instrument are NOT applicable to the modified score and have therefore been omitted here; they are reproduced for reference in Supplementary Table S3.

Original FINDRISC Band	Score	n (%)
Very Low	<7	1403 (83.3%)
Low	7–11	271 (16.1%)
Moderate	12–14	10 (0.6%)
High	15–20	0 (0.0%)
Very High	>20	0 (0.0%)
**Mean score (SD)**		4.32 (2.59)

#### 3.2.3. MUO (Metabolically Unhealthy Obesity) Score Distribution

**Phenotype Identification:**

**Phenotype**

**Definition**

**n (%)**

**Interpretation**
Metabolically HealthyVFL < 7, normal range979 (58.1%)Low intervention priorityBIA-Defined TOFI-Like (Hidden Risk)BMI < 25 AND VFL ≥ 1087 (5.2% of cohort)Not identified by BMI aloneSarcopenic-Obesity-Like PhenotypePBF ≥ 30% AND SMM < 30 kg (M)/23 kg (F)166 (9.9%)Operational rule (ii) of Section 2.2.3; not equivalent to EWGSOP2/ESPEN–EASO criteriaRisk-Enriched Visceral-Fat/BPVFL ≥ 10 AND SBP ≥ 130151 (9.0%)May warrant further evaluation in prospective studies


BIA-defined TOFI-like detection: among the 1187 normal-weight participants (BMI < 25 kg/m^2^), 87 were identified as having a BIA-defined TOFI-like phenotype (thin outside, fat inside), that is, a normal BMI combined with elevated visceral fat (VFL ≥ 10). This corresponds to 5.2% of the full cohort (95% CI 4.2–6.3%) and to 7.3% of all normal-weight participants (87/1187). Because visceral fat was assessed by bioelectrical impedance rather than by CT or MRI, the phenotype is described operationally as BIA-defined TOFI-like. These individuals would be classified as normal weight by BMI alone, yet show a body-composition pattern associated with elevated visceral adiposity.

This finding underscores the limitation of BMI-only screening and supports the integration of DSM-BIA body composition assessment, since an adverse body-composition profile (elevated MUO score) was observed in a substantial fraction of normal-weight participants. In addition, 151 participants (9.0%) met a combined risk-enriched visceral-fat/BP phenotype, defined by elevated visceral fat (VFL ≥ 10) together with a systolic blood pressure ≥ 130 mmHg (Table 4); this readily screenable subgroup, in whom visceral adiposity and elevated blood pressure co-occur, may warrant further evaluation in prospective studies.

**Table 4:** MUO score distribution and phenotype classification (N = 1684).

MUO Category	Score	Interpretation	n (%)
Low Risk	<30	Routine follow-up	997 (59.2%)
Moderate Risk	30–60	Candidate for lifestyle counselling	320 (19.0%)
High Risk	≥60	Risk-enriched phenotype, potential target for early intervention	367 (21.8%)
**Mean MUO score (SD)**			27.22 (27.56)

### 3.3. Correlation Analysis

Before presenting these associations, we restate the structural dependency described in Section 2.6. The MUO score is computed from visceral fat level, percent body fat, skeletal muscle mass and systolic blood pressure, and the patient similarity network uses both these variables and the composite scores derived from them. Correlations among them therefore quantify construct consistency—the degree to which a composite behaves as its own definition implies—and are not independent biological validation. Associations between two separately measured DSM-BIA parameters, such as visceral fat level and percent body fat, are not affected by this dependency and are interpretable as empirical associations.

**Figure 4:**
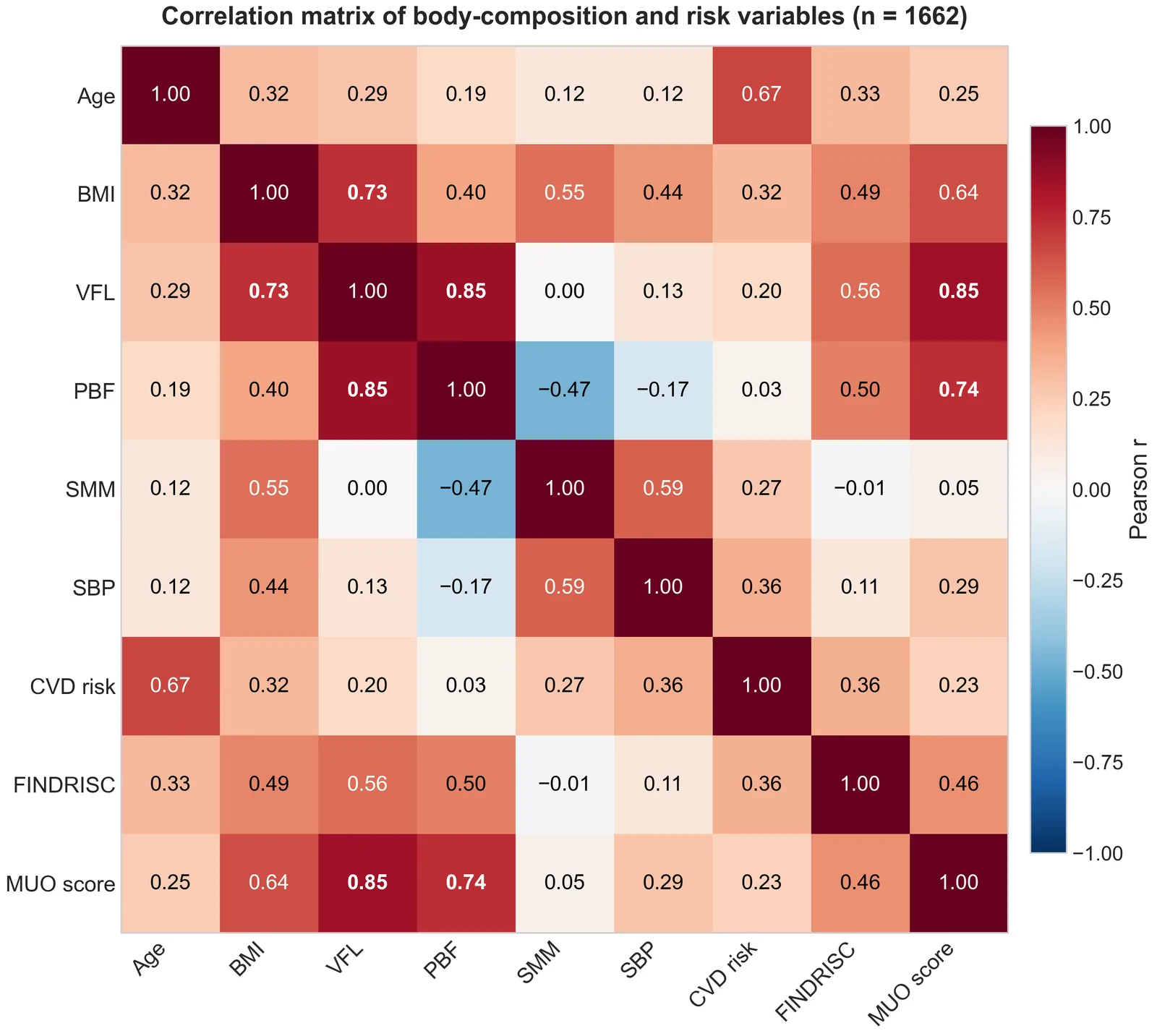
Pearson correlation matrix (full symmetric form) between anthropometric, body-composition and risk-score variables (complete-case n = 1662). Cell values are Pearson product-moment coefficients (r); pairwise *p*-values were adjusted for multiple comparisons using the Benjamini–Hochberg false discovery rate (FDR) procedure, and the pattern was confirmed with Spearman rank correlations. Abbreviations: BMI, body mass index; VFL, visceral fat level; PBF, percent body fat; SMM, skeletal muscle mass; SBP, systolic blood pressure; CVD Risk, 10-year cardiovascular disease risk; FINDRISC, modified Finnish Diabetes Risk Score; MUO, Metabolically Unhealthy Obesity score.

**Figure 5:**
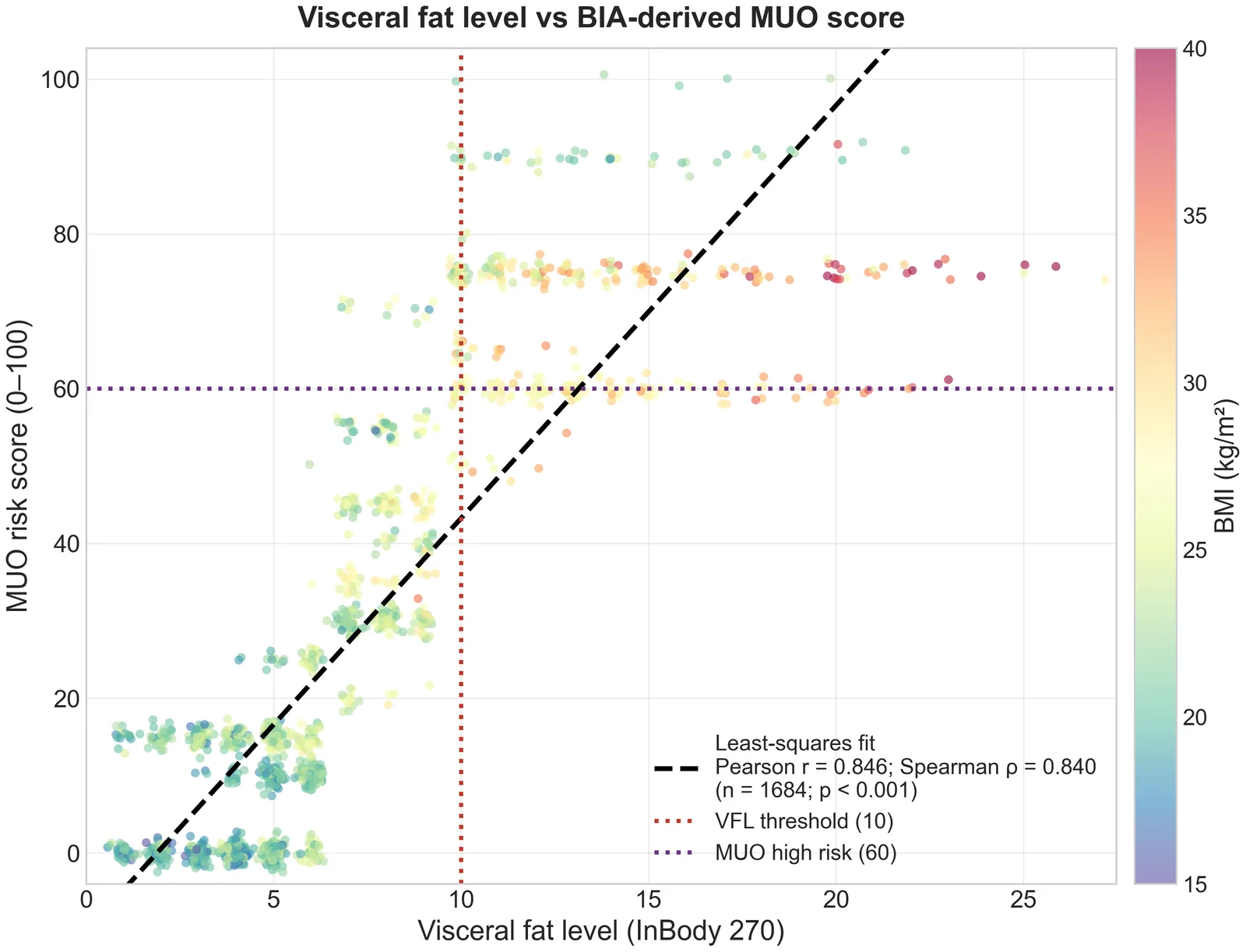
Relationship between visceral fat level and the Metabolically Unhealthy Obesity (MUO) score (Pearson r = 0.846; Spearman rho = 0.840; n = 1684). Because the MUO score is a discrete composite that includes visceral fat level as a component, both Pearson and Spearman coefficients are reported, and the association reflects internal construct consistency rather than independent validation. Points are coloured by body mass index; small random jitter was added to separate overlapping discrete values. Dashed line, least-squares fit; dotted lines, the VFL ≥ 10 and MUO ≥ 60 thresholds.

Key Finding: Visceral fat level correlated very strongly with percent body fat (r = 0.855, *p* < 0.001; Figure 4; Table 5) and with the MUO score (r = 0.846, *p* < 0.001; Figure 5). The VFL-MUO association partly reflects the construction of the MUO score, in which visceral fat level is a major component; it should therefore be interpreted as internal consistency within the defined construct rather than independent external validation.

**Table 5:** Principal Pearson correlation coefficients between key variables (N = 1684; n = 1662 where SBP or CVD risk is involved; Benjamini–Hochberg FDR-adjusted *p*-values; the pattern was confirmed with Spearman correlations). Only the most informative coefficients are shown; the complete pairwise correlation matrix, with Spearman coefficients and 95% confidence intervals for all 45 pairwise associations, is provided in Supplementary Table S5. Pairs whose members share components by construction are flagged in the final column.

Variable 1	Variable 2	r	*p*-Value	Interpretation/Dependency
VFL	PBF	0.855	<0.001	Very strong positive
VFL	MUO Score	0.846	<0.001	Very strong positive—shares components (construct consistency)
BMI	VFL	0.728	<0.001	Strong positive
Age	CVD Risk	0.666	<0.001	Strong positive
VFL	FINDRISC	0.558	<0.001	Strong positive
PBF	CVD Risk	0.032	0.197	Non-significant
VFL	SMM	−0.004	0.879	Non-significant

Abbreviations: BMI (body mass index); VFL (visceral fat level); PBF (percent body fat); SMM (skeletal muscle mass); SBP (systolic blood pressure); CVD (cardiovascular disease); FINDRISC (modified Finnish Diabetes Risk Score); MUO (Metabolically Unhealthy Obesity).

By contrast, the VFL-PBF correlation links two independently measured DSM-BIA parameters and is not subject to this circularity. Notably, the PBF-CVD correlation was non-significant (r = 0.032, *p* = 0.197), reflecting that the Office-Based Framingham equation does not directly incorporate body-fat parameters and is dominated by age and sex. This supports the complementary nature of integrating the scoring systems, as they capture distinct aspects of cardiometabolic risk. All correlation *p*-values were adjusted for multiple comparisons using the Benjamini–Hochberg false discovery rate procedure, and the ranking of associations was confirmed with Spearman correlations.

### 3.4. Sex-Stratified Analysis

**Figure 6:**
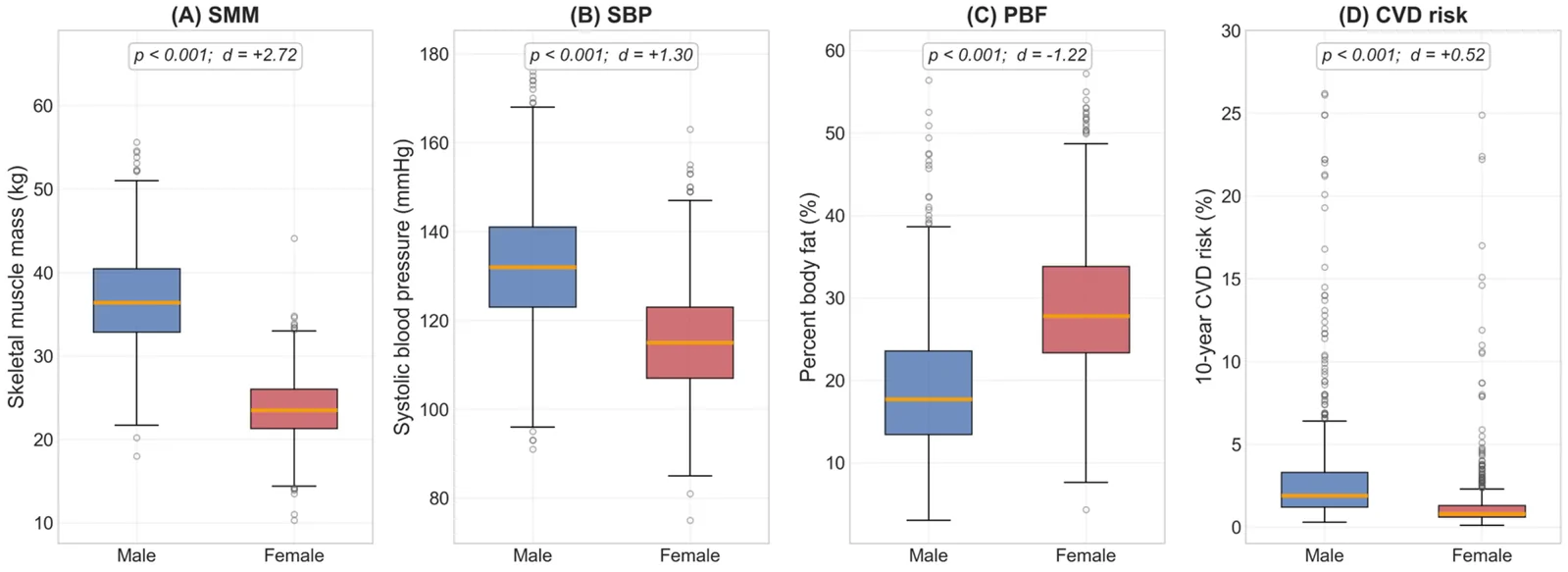
Sex-stratified comparison of four key cardiometabolic parameters (male n = 839, female n = 845): (**A**) skeletal muscle mass (SMM); (**B**) systolic blood pressure (SBP); (**C**) percent body fat (PBF); and (**D**) 10-year cardiovascular disease (CVD) risk. Boxes show medians and interquartile ranges; whiskers extend to 1.5× IQR and circles denote outliers. Between-sex differences were assessed with Welch’s *t*-tests; all four comparisons were significant at *p* < 0.001. The corresponding Cohen’s d effect sizes (Table 6) were large for skeletal muscle mass (SMM, d = 2.72), systolic blood pressure (SBP, d = 1.30) and percent body fat (PBF, d = −1.22), and medium for 10-year cardiovascular disease (CVD) risk (d = 0.52).

Significant sex differences were observed across most parameters (Figure 6; Table 6). Males demonstrated significantly higher CVD risk (d = 0.52), BMI (d = 0.65), systolic blood pressure (d = 1.30) and skeletal muscle mass (d = 2.72, reflecting expected physiological sex differences). Females showed significantly higher percent body fat (d = −1.22), visceral fat level (d = −0.27) and FINDRISC (d = −0.43).

**Table 6:** Sex-stratified risk score comparison (Welch *t*-tests + Cohen’s d).

Variable	Male (n = 839)	Female (n = 845)	*p*-Value	Cohen’s d
CVD Risk, %, mean (SD)	2.95 (4.14)	1.26 (1.97)	<0.001	0.52 (medium)
MUO Score, mean (SD)	26.87 (26.37)	27.56 (28.70)	0.607	−0.03 (negligible, ns)
VFL, mean (SD)	6.40 (4.44)	7.59 (4.23)	<0.001	−0.27 (small)
PBF (%), mean (SD)	19.18 (7.97)	28.94 (7.99)	<0.001	−1.22 (large)
SMM (kg), mean (SD)	36.82 (5.80)	23.66 (3.64)	<0.001	2.72 (large)
BMI, kg/m^2^, mean (SD)	24.81 (4.13)	22.23 (3.83)	<0.001	0.65 (medium)
SBP (mmHg), mean (SD)	132.54 (13.71)	115.47 (12.60)	<0.001	1.30 (large)
DBP (mmHg), mean (SD)	78.16 (9.42)	75.88 (8.79)	<0.001	0.25 (small)
FINDRISC, mean (SD)	3.77 (2.85)	4.86 (2.18)	<0.001	−0.43 (small)

Notably, the composite MUO score did **not** differ significantly between sexes (26.87 vs. 27.56; *p* = 0.607, d = −0.03), indicating that although the distribution of adiposity differs by sex (males: higher central CVD burden; females: higher overall body-fat percentage), the overall body-composition risk burden captured by the MUO construct was comparable. This reflects the sex-specific PBF thresholds embedded in the MUO algorithm, which calibrate body-fat scoring to physiological norms.

### 3.5. Age Subgroup Analysis

Given the wide age range, we compared cardiometabolic parameters across four age groups (≤25, 26–35, 36–50, >50 years) using one-way ANOVA with η^2^ effect sizes (Table 7).

**Table 7:** Age-group one-way ANOVA (≤25, 26–35, 36–50, >50 years).

Variable	≤25	26–35	36–50	>50	η^2^	*p*-Value
CVD Risk, %	1.42	2.49	6.22	15.92	0.506	<0.001
FINDRISC	4.02	4.39	6.76	9.28	0.142	<0.001
BMI, kg/m^2^	22.96	25.83	26.85	27.22	0.094	<0.001
VFL	6.50	8.45	10.20	11.74	0.076	<0.001
MUO Score	24.58	35.33	44.02	52.95	0.055	<0.001
PBF, %	23.41	25.24	29.10	32.39	0.039	<0.001
SMM, kg	29.77	33.46	32.05	29.49	0.020	<0.001
SBP, mmHg	123.47	125.52	125.38	132.50	0.009	0.002

Age exerted by far the strongest effect on 10-year CVD risk (η^2^ = 0.506) and FINDRISC (η^2^ = 0.142), consistent with the age weighting of both instruments. Body-composition markers (BMI, VFL, MUO, PBF) increased monotonically with age, confirming that visceral adiposity accumulates progressively even within this predominantly young cohort.

**MUO Score by BMI Category:**

The MUO score increased strongly across BMI categories, from normal-weight through overweight to obese participants, with the obese group showing the highest mean MUO scores. This monotonic gradient is what the construct is designed to produce, and it therefore demonstrates construct consistency against conventional anthropometric categories rather than independent biological validation. It also reveals a substantial body-composition burden within the normal-weight stratum (the BIA-defined TOFI-like subgroup).

### 3.6. Multivariable Models and Score Concordance

We examined associations between participant features and three binary outcomes (Table 8). Outcome C (the BIA-defined TOFI-like phenotype, 87 events) was modelled with multivariable logistic regression. Outcomes A and B had very few events (41 and 10, respectively); for these, Firth’s penalised logistic regression was used, and the results are reported strictly as exploratory, descriptive feature associations rather than as predictive models, since fewer than approximately ten events per variable cannot support a valid multivariable prediction model.

**Table 8:** Feature associations for risk outcomes (Model C multivariable; Models A and B exploratory/descriptive only).

Model	Outcome	Method	Events (n, %)	AUC †	Associated Features (OR)
A (exploratory)	High CVD risk (≥10%)	Firth penalised logistic	41 (2.4%)	0.995 †	Age 1.40; male sex 17.2; smoker 10.2
B (exploratory)	Elevated modified FINDRISC (≥12)	Firth penalised logistic	10 (0.6%)	0.999 †	Age 1.35
C	BIA-defined TOFI-like phenotype	Multivariable logistic (McFadden R^2^ = 0.417)	87 (5.2%)	0.93 (5-fold CV; 0.938 in-sample)	PBF 1.20; SMM 0.859; age 0.962

† Due to the very low event counts for outcomes A and B, the near-perfect AUC values reflect in-sample separation rather than true predictive discrimination, and should be viewed strictly as descriptive feature associations. Only Model C (87 events) provides enough events to support a cautious multivariable interpretation, and even this requires external validation. In stratified five-fold cross-validation, Model C retained an out-of-fold AUC of 0.93 (in-sample 0.938), indicating limited apparent optimism. However, this high apparent discrimination likely reflects overlap between predictor variables and the definition of the BIA-defined TOFI-like phenotype, as several predictors are strongly correlated with its defining components. Accordingly, the reported performance should be interpreted as reflecting internal separability within the feature space rather than generalizable predictive accuracy.

**Score Concordance.** Agreement between the three non-invasive risk constructs (dichotomized at their elevated-risk thresholds) was low across all pairings:
**Pair****Cohen’s κ****Lin’s CCC**Framingham CVD vs. MUO0.0680.031FINDRISC vs. MUO−0.0880.050Framingham CVD vs. FINDRISC−0.0300.273

The uniformly low concordance (κ near 0; CCC ≤ 0.27) indicates that the three instruments capture **complementary** rather than redundant dimensions of cardiometabolic risk, justifying their combined use in a multi-construct screening framework.

### 3.7. Patient Similarity Network Analysis

**Figure 7:**
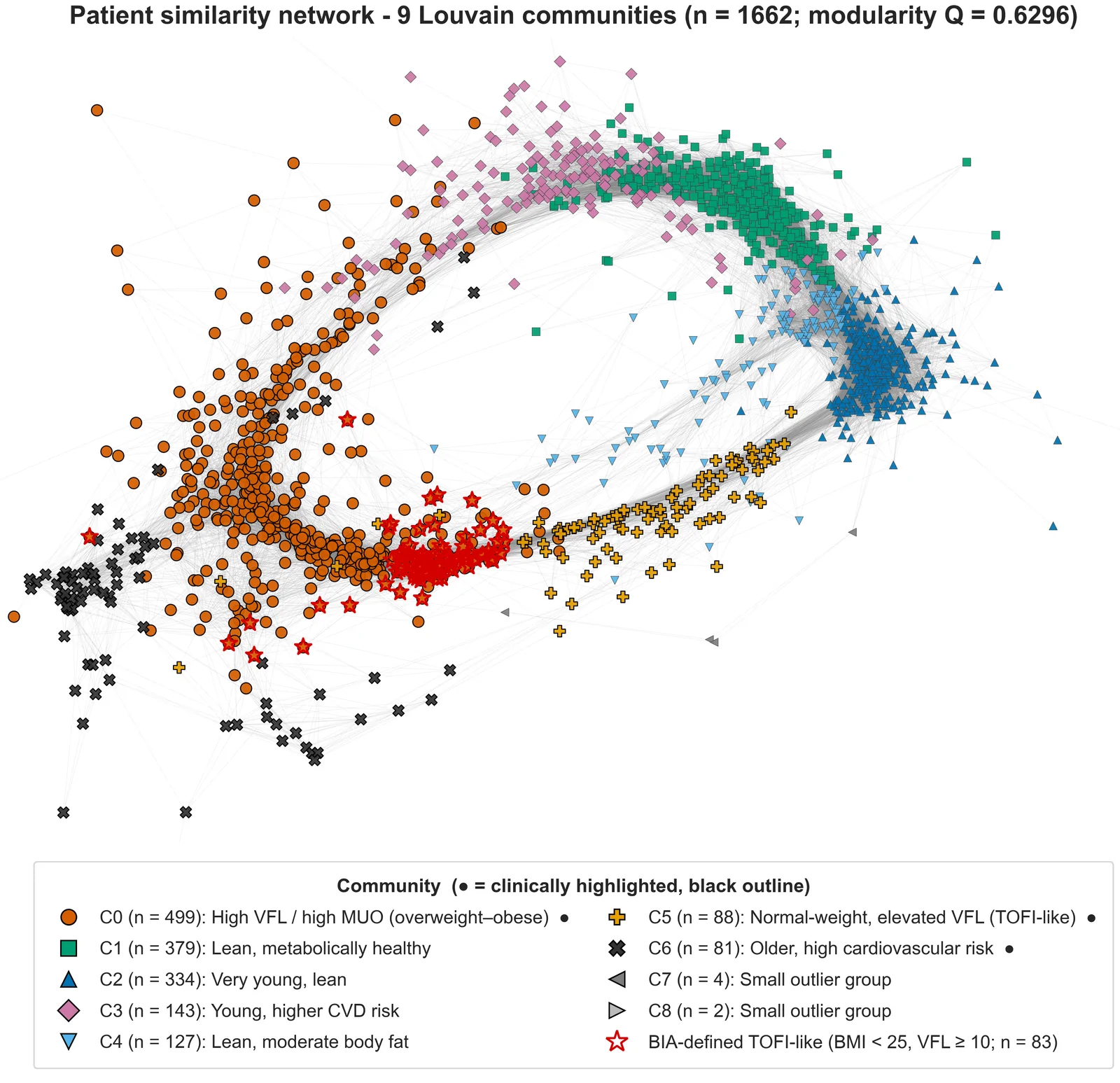
Patient similarity network of the cohort (N = 1662 participants with a complete feature vector). Each node is a participant, positioned by a force-directed (Fruchterman–Reingold) layout and coloured by Louvain community (modularity Q = 0.6296; resolution γ = 1.0, seed = 42); the five isolated nodes (no supra-threshold similarity link) are not shown. The nine communities correspond to the phenotypes summarised in Table 10; the highlighted communities C0 (high visceral fat/high MUO), C5 (normal-weight with elevated visceral fat, BIA-defined TOFI-like) and C6 (older, high cardiovascular risk) are marked with black outlines, and BIA-defined TOFI-like hidden-risk participants (BMI < 25 with VFL ≥ 10) are overlaid as red stars. VFL, visceral fat level; MUO, Metabolically Unhealthy Obesity; TOFI, thin outside fat inside.

The patient similarity network was constructed on the 1662 participants with a complete feature vector, including blood pressure (Figure 7). Network statistics are given in Table 9.

**Table 9:** Network statistics.

Metric	Value
Total nodes (participants)	1662
Total edges (similarity links)	69,015
Network density	0.0500
Target density ($\rho_{target}$)	0.05
Adaptive similarity threshold (τ)	0.832
Average clustering coefficient	0.5794
Average node degree	83.05
Number of communities (Louvain, unweighted, γ=1.0, seed = 42)	9
Modularity (Q)	0.6296
Configuration-model null Q, mean (SD)	0.0813 (0.0016)
Permutation *p*-value (Q_obs vs. null)	0.005

The observed modularity (Q=0.6296) vastly exceeded the degree-preserving configuration-model null (Q=0.0813±0.0016; permutation p=0.005), confirming that the nine-community structure is far stronger than expected by chance.

Of the 1662 networked participants, 1657 were assigned to one of the nine Louvain communities; the remaining five were isolated nodes (degree 0 at the adaptive similarity threshold τ=0.832) and were therefore excluded from the community partition. Accordingly, the per-community counts in Table 10 sum to 1657 (the rounded percentages sum to 99.6%).

**Table 10:** Participant community characteristics (Louvain detection, unweighted, γ=1.0, seed = 42).

Cluster	n (% of 1662)	Mean Age	Mean BMI	Mean VFL	Mean CVD%	Mean FINDRISC	Mean MUO	Phenotype
C0	499 (30.0%)	23.6	27.6	11.8	2.17	5.8	61.2	High VFL/high-MUO (overweight–obese)
C1	379 (22.8%)	21.0	21.8	3.5	1.60	1.4	7.6	Lean, metabolically healthy
C2	334 (20.1%)	18.5	19.9	4.6	0.85	5.1	4.8	Very young, lean
C3	143 (8.6%)	18.9	24.0	4.4	2.70	4.9	11.7	Young, higher-CVD
C4	127 (7.6%)	22.9	20.9	5.8	0.66	1.6	16.1	Lean, moderate body fat
C5	88 (5.3%)	19.6	22.4	7.8	0.92	5.5	34.9	Normal-weight, elevated visceral fat (BIA-defined TOFI-like)
C6	81 (4.9%)	48.3	25.9	8.6	11.66	7.4	39.8	Older, high cardiovascular risk
C7	4 (0.2%)	20.2	24.0	7.2	0.82	6.2	20.0	Small outlier group
C8	2 (0.1%)	32.0	22.9	4.5	1.80	5.0	0.0	Small outlier group

Phenotypic interpretation: The network resolved nine communities—seven phenotypically interpretable subgroups plus two small residual outlier groups (C7, C8). The largest community (C0, 30.0%) captured overweight–obese participants with high visceral fat and high MUO scores. Community C5 represents a BIA-defined TOFI-like subgroup characterised by near-normal mean BMI (22.4) but elevated visceral fat (7.8) and elevated MUO (34.9). Community C6, although small (4.9%), isolated older participants with elevated cardiovascular risk (mean age 48.3 years, mean 10-year CVD risk 11.7%). The two smallest groups (C7, C8) represent residual outlier clusters.

These results show that network-based clustering identified phenotypically distinct subgroups within the multidimensional feature space that BMI alone would not separate. These groupings arise from the structure of the input variables and their weighting scheme, and therefore should not be interpreted as externally validated participant subtypes.

#### 3.7.1. Comparison with Conventional Clustering and Dimensionality-Reduction Approaches

To establish whether the network representation offers anything beyond established multivariate techniques, we applied k-means clustering, Ward agglomerative clustering, principal component analysis (PCA; three components retaining ≥ 80% of the variance) followed by k-means, and Uniform Manifold Approximation and Projection (UMAP) embedding followed by Hierarchical Density-Based Spatial Clustering of Applications with Noise (HDBSCAN) to the identical weighted, standardised nine-feature matrix. Methods that require the number of clusters to be fixed in advance were matched to the nine communities recovered by Louvain. Full results are given in Supplementary Table S6. Three findings emerged.

First, the phenotypic axes recovered by the network are not an artefact of the graph representation. The k-means and Ward partitions agreed substantially with the Louvain partition (adjusted Rand index, ARI, 0.50 and 0.42; normalised mutual information, NMI, 0.63 and 0.60), and PCA followed by k-means agreed similarly (ARI 0.40; NMI 0.57). Convergence across three independent method families indicates that the principal body-composition contrasts described in Table 10 are properties of the data rather than of the algorithm.

Second, no single method dominated. The centroid-based methods scored higher on the internal indices that they themselves optimise (silhouette 0.18–0.22 versus 0.05 for the network partition; Calinski–Harabasz 427–496 versus 239), and k-means concentrated the BIA-defined TOFI-like participants into a single cluster more tightly than the network did (best-cluster F1 0.60 versus 0.29). Conversely, the network isolated the small older, high-cardiovascular-risk subgroup better than any comparator (best-cluster F1 0.70 versus 0.54–0.62). Reproducibility under 80% subsampling was broadly comparable (mean pairwise ARI 0.73 for the network; 0.56–0.83 for the comparators).

Third, and most relevant to the choice of method, every centroid-based approach requires the number of clusters to be fixed a priori. Applying the conventional silhouette criterion to k-means selected k = 2, which collapses the cohort into two coarse strata and dissolves precisely the small subgroups of interest—the older high-cardiovascular-risk participants and the normal-weight participants with elevated visceral fat. UMAP followed by HDBSCAN, the only comparator that also determines the number of clusters from the data, returned four clusters, left 24 participants unassigned, produced a negative silhouette in the original feature space (−0.13) and recovered neither reference subgroup (best-cluster F1 ≤ 0.12).

This benchmark does not bear on H5, which concerns whether the network’s own partition is statistically robust and phenotypically interpretable, and not whether it is superior to alternative algorithms. The comparison is reported because the choice of a network representation should be justified rather than assumed, and it is reported in full, including the criteria on which the comparators perform better.

### 3.8. Computational Performance

The computational perfomance of the framework, including the time complexity and measured execution times for each processing step, is summarized below.



**Operation**

**Time Complexity**

**Measured Time (N = 1684)**
Risk calculation per participantO(1)<50 msSimilarity matrix constructionO(n^2^)~12 sLouvain community detectionO(n log n)~1 sFull analysis pipeline-<60 s


### 3.9. Hypothesis Evaluation of the Framework

Bringing together the preceding results, we evaluated each of the six predefined hypotheses (Section 1.4) against their confirmation criterion (Table 11). All six hypotheses were supported, indicating that the framework performed as designed across every evaluated dimension—hidden-risk detection, construct consistency, complementarity, discrimination, network stratification and sex-calibration.

**Table 11:** Evaluation of the six predefined hypotheses against the framework’s results.

Hypothesis	Prediction	Key Supporting Result	Verdict
H1—Hidden-risk detection	Detects risk-enriched phenotypes not captured by BMI	BIA-defined TOFI-like 5.2% of cohort (7.3% of normal-weight); high MUO 21.8%; sarcopenic-obesity-like 9.9%; combined risk-enriched visceral-fat/BP phenotype 9.0%	Supported
H2—Construct consistency/dose–response	Risk rises monotonically with BMI and age	MUO and VFL increase monotonically across BMI categories and age groups (ANOVA *p* < 0.001; VFL η^2^ = 0.076, MUO η^2^ = 0.055)	Supported
H3—Complementarity	Three constructs are non-redundant	Cohen’s κ −0.088 to 0.068; Lin’s CCC 0.031–0.273 across all pairings	Supported
H4—Discrimination	Model discriminates the hidden-risk phenotype	BIA-defined TOFI-like model cross-validated AUC = 0.93 (in-sample 0.938), McFadden R^2^ = 0.417 (PBF OR 1.20, SMM OR 0.859, age OR 0.962)	Supported
H5—Network stratification validity	Network recovers robust, reproducible strata	Modularity Q = 0.6296 vs. null 0.0813 ± 0.0016 (permutation *p* = 0.005); deterministic seed = 42; nine communities incl. BIA-defined TOFI-like (C5) and older high-CVD (C6)	Supported
H6—Sex-calibration invariance	Composite burden is sex-invariant	MUO 26.87 (M) vs. 27.56 (F), *p* = 0.607, d = −0.03, despite large sex differences in PBF (d = −1.22), SMM (d = 2.72), SBP (d = 1.30)	supported

Because the framework was evaluated on cross-sectional data, these six confirmations provide preliminary evidence of internal construct consistency, internal discrimination and phenotypic separability—that is, evidence that the framework behaves as designed. They do not, by themselves, establish prospective outcome-predictive accuracy, which would require the longitudinal validation outlined in Section 4.8.

## 4. Discussion

### 4.1. Principal Findings

This study set out to evaluate whether a non-invasive clinical framework for cardiometabolic risk stratification operates consistently with its predefined design in a young adult population. The six predefined hypotheses (H1–H6) provide a structured and falsifiable approach to assessing internal framework behaviour. The framework integrates Office-Based Framingham risk scoring, a modified FINDRISC, a BIA-derived MUO score, InBody 270 DSM-BIA body-composition phenotyping and a weighted patient similarity network. All six hypotheses were supported (Section 3.9, Table 11). Several key findings underpin this conclusion:

Hypothesis Confirmation: Each predefined hypothesis met its confirmation criterion (Table 11). The framework detected hidden-risk phenotypes beyond BMI (H1), reproduced established BMI- and age-related risk gradients (H2), combined non-redundant risk constructs (H3), discriminated the BIA-defined TOFI-like phenotype with high internal feature-space separability (H4), recovered a statistically robust, reproducible network partition (H5), and remained sex-calibrated despite large sex differences in its body-composition inputs (H6). Taken together, these results provide convergent, hypothesis-driven evidence that the framework organises multiple risk-related features into internally consistent and interpretable patterns, in line with its predefined analytical framework.

Early Risk Identification: Despite the young mean age (22.5 years, median 20), 21.8% of participants demonstrated high MUO scores and 5.2% of the full cohort (7.3% of normal-weight participants) exhibited a BIA-defined TOFI-like phenotype (normal weight but elevated visceral fat). This supports the rationale for early, hypothesis-generating screening before clinical disease manifestation, although prospective follow-up is required to confirm prognostic relevance. A further 9.0% of participants displayed a combined risk-enriched visceral-fat/BP phenotype (VFL ≥ 10 and systolic blood pressure ≥ 130 mmHg), in whom elevated visceral adiposity and blood pressure co-occurred and which is readily identifiable at the point of screening.

BIA-Defined TOFI-Like Detection: The identification of 87 BIA-defined TOFI-like individuals (normal BMI but elevated bioimpedance-derived visceral fat) is a potentially relevant finding. These participants would be classified as “healthy weight” by conventional screening but show a body-composition pattern associated with elevated metabolic risk. Because visceral fat was not confirmed by CT or MRI, this phenotype is described as BIA-defined TOFI-like and requires imaging-based and longitudinal confirmation.

Sex Differences: Males exhibited higher 10-year CVD risk, BMI and systolic blood pressure, while females had higher percent body fat and visceral fat level; the composite MUO score did not differ significantly between sexes (*p* = 0.607). This reflects the different risk determinants captured by each instrument: the Office-Based Framingham equation heavily weights male sex, whereas the sex-calibrated MUO score balances body-fat distribution patterns that differ physiologically between sexes.

Network Clustering Structure: The nine identified participant communities (seven phenotypically interpretable subgroups plus two small residual outlier groups) showed a strong, statistically robust structure (modularity Q = 0.6296 versus a configuration-model null of 0.0813 ± 0.0016; permutation *p* = 0.005). The Louvain algorithm with a deterministic seed (42) and resolution γ = 1.0 was applied to the unweighted similarity graph over connected vertices, producing well-separated, interpretable communities ranging from lean, metabolically healthy young adults to an older, high-cardiovascular-risk subgroup. Because the similarity network was built in part from composite scores (MUO, Office-Based Framingham, modified FINDRISC) that themselves incorporate age, BMI and VFL, this structure reflects internally consistent phenotyping rather than independent external validation; the modularity statistic quantifies the strength of the partition, not its clinical predictive value.

### 4.2. Comparison with Literature

Our findings are consistent with the broader literature on visceral adiposity and body-composition phenotyping. The BIA-defined TOFI-like prevalence of 5.2% (n = 87/1684; 7.3% of normal-weight participants) lies within the 5–10% range reported for metabolically unhealthy normal-weight and normal-weight central-obesity phenotypes in general adult populations [36,37,38,39,40,41,42]. The dominant role of visceral fat in our correlation and network analyses is consistent with imaging-based and mechanistic evidence that visceral and ectopic fat, rather than BMI, drive insulin resistance and cardiometabolic risk [43,44,45,46,47,48,49]. Because we did not have longitudinal outcomes, we deliberately refrain from reporting predictive-accuracy metrics; the network modularity (Q = 0.6296 vs. configuration-model null Q = 0.0813, *p* = 0.005) quantifies the strength of the phenotypic partition rather than its prognostic value, and all associations require prospective, longitudinal validation.

### 4.3. Methodological Positioning: Why a Patient Similarity Network?

The benchmark reported in Section 3.7.1 clarifies what the network representation does and does not contribute. Conventional clustering optimises compactness in a Euclidean feature space and, unsurprisingly, outperforms a graph partition on indices constructed from that same criterion; we therefore do not claim superior cluster quality. The specific contributions of the network are threefold. First, granularity is inferred rather than imposed: conventional selection of k by the silhouette criterion would have returned two clusters, merging precisely the small subgroups that motivate screening—older participants with elevated cardiovascular risk, and normal-weight participants with elevated visceral fat. Second, modularity supports an explicit inferential test of whether the observed structure exceeds chance, by comparison with a degree-preserving configuration-model null; centroid-based methods offer no equivalent test, so a k-means partition of structureless data is not formally distinguishable from a k-means partition of real structure. Third, the graph retains the continuity between subgroups, which suits a young screening cohort in which cardiometabolic risk is distributed along gradients rather than in discrete diagnostic classes; the force-directed layout in Figure 7 displays the phenotypes as a connected continuum rather than as isolated islands. The substantial agreement between the network and the centroid-based partitions (ARI 0.40–0.50) is itself informative, because it indicates that the phenotypic organisation described here is a property of the data. Conversely, the failure of UMAP followed by HDBSCAN to recover either reference subgroup shows that nonlinear embedding is not automatically preferable in this setting. We therefore present the patient similarity network as a stratification approach with specific, stated advantages, and not as a demonstrated improvement in risk prediction, which would require longitudinal outcome data.

### 4.4. Biological Interpretation of the Identified Phenotypes

The phenotypes surfaced by the framework are interpretable in the light of established adipose-tissue biology. Visceral adipose tissue is functionally distinct from subcutaneous fat: it is more lipolytic, less responsive to the antilipolytic action of insulin, and drains directly into the portal circulation, exposing the liver to an elevated flux of free fatty acids and adipokines [43,46]. The resulting ectopic lipid deposition in liver and skeletal muscle, rather than total fat mass, is the proximate correlate of insulin resistance, which is why individuals matched for BMI can differ markedly in metabolic risk [40,44,48]. The strong association we observed between visceral fat level and percent body fat (r = 0.855), together with the near-null association between visceral fat level and skeletal muscle mass (r = −0.004), is consistent with this compartment-specific view of adiposity.

The BIA-defined TOFI-like phenotype corresponds to what has been described as the metabolically obese, normal-weight individual [38] and, more recently, as metabolically unhealthy normal weight [41]. Its proposed basis is a limited capacity for safe subcutaneous fat expansion—determined by adipocyte hyperplasia, sex, genetic background and physical activity—so that when energy surplus exceeds that capacity, lipid is deposited viscerally and ectopically at a relatively low total body weight [37,41]. Magnetic resonance phenotyping has confirmed that a considerable proportion of normal-weight adults carry an internal fat burden comparable to that of overweight individuals [37,40]. This mechanism explains why a BMI-based screen is structurally unable to detect this group, and why a body-composition measurement is required to reveal it.

The sarcopenic-obesity-like phenotype identified in 9.9% of participants reflects the second axis of the muscle–fat relationship. Skeletal muscle is the principal site of insulin-stimulated glucose disposal, so low muscle mass combined with high fat mass compounds metabolic risk beyond the effect of adiposity alone; current consensus definitions accordingly require both components [27,28]. Its appearance in a cohort with a median age of 20 years is notable, because sarcopenic obesity is usually conceptualised as a disorder of ageing. Whether these young individuals represent early muscle underdevelopment rather than muscle loss cannot be resolved from cross-sectional data.

The sex differences we observed follow the expected physiology of fat distribution. Women store proportionally more lipid subcutaneously, particularly in the gluteofemoral depot, which is associated with a comparatively favourable metabolic profile, whereas men accumulate proportionally more visceral fat at any given BMI [47]. The higher percent body fat in women, alongside the higher blood pressure, skeletal muscle mass and Framingham ordering in men and the absence of a significant difference in the composite MUO score, is consistent with this pattern and with the sex-specific percent-body-fat thresholds embedded in the MUO construct. It also underlines that a single adiposity threshold applied across both sexes would misclassify risk in both directions. Finally, the monotonic increase in visceral fat level and MUO score across age groups within this predominantly young cohort is consistent with the progressive redistribution of fat towards visceral and ectopic depots described in imaging studies, and with the observation that central adiposity predicts mortality independently of BMI [45,49].

These interpretations are mechanistic hypotheses generated by the framework, not findings of the present study. None of the underlying processes—portal free fatty acid flux, ectopic lipid deposition, adipose-tissue expandability or insulin-mediated glucose disposal—was measured here, and establishing them would require the laboratory and imaging measurements listed in Section 4.8.

### 4.5. Strengths

Non-Invasive Approach: Complete risk stratification without laboratory values (lipids, glucose), using only office-based measurements and DSM-BIA.

Large Sample Size: N = 1684 participants with complete body composition data provides robust statistical power (minimum detectable r ≈ 0.048, d ≈ 0.135).

BIA-Defined TOFI-Like Detection: Capability to identify body-composition phenotypes not identified by BMI alone.

Established Risk Constructs: Integration of the Office-Based Framingham equation [11] and a modified, proxy-based FINDRISC, applied transparently with their documented assumptions and limitations.

DSM-BIA Technology: InBody 270 Direct Segmental Multi-frequency measurement provides rapid, non-invasive body-composition assessment; InBody-derived body-composition and abdominal-fat estimates have shown acceptable agreement with dual-energy X-ray absorptiometry (DXA) and magnetic resonance imaging (MRI) reference methods in independent validation studies [50,51,52,53].

Methodological Transparency and Benchmarking: the network partition was compared head-to-head with k-means, Ward hierarchical, PCA-based and UMAP-based clustering on the identical feature matrix (Section 3.7.1; Supplementary Table S6), and the analysis code, seeds and thresholds are reported in full, so that the partition can be reproduced exactly.

### 4.6. Limitations

Cross-Sectional Design: Single time-point data without longitudinal outcome validation prevents assessment of predictive accuracy.

Young Population: Mean age 22.5 years (median 20) limits generalizability to older populations where CVD/T2DM risk is higher.

Proxy Variables: Self-reported smoking status and the antihypertensive-medication proxy may introduce misclassification; first-degree family history of diabetes was not systematically recorded and was scored conservatively as zero (see the FINDRISC limitation below).

Single-Centre: Data derive from a single national screening programme without an independent external validation cohort; the sample may not be representative of all Hungarian young adults, and external, multi-cohort replication is required.

No Outcome Data: Without CVD/T2DM outcome events, true predictive performance cannot be calculated; accordingly, the cross-validated AUC for the BIA-defined TOFI-like classification reflects internal feature-space separability of an operationally defined phenotype and is not equivalent to clinical prediction.

BIA-derived visceral fat is an indirect proxy: Importantly, BIA-derived VFL is an indirect proxy and not equivalent to visceral fat volume measured by CT or MRI [54]. Furthermore, BIA measurements can be influenced by hydration status, recent physical activity, food intake and, in women, menstrual cycle phase. The lack of standard laboratory parameters (lipid panel, HbA1c) also restricts the diagnostic capability of our models, which should be regarded as hypothesis-generating.

Off-label risk scoring in young adults: The Office-Based Framingham equation was derived and validated in adults aged 30–74 years; its application to participants under 30 years is exploratory and off-label, so absolute CVD risks in the youngest participants reflect relative ordering rather than calibrated 10-year probabilities. We nevertheless retained this model because the alternatives validated in younger adults (e.g., QRISK3 [4], SCORE2 [55]) all require laboratory lipid measurements that are unavailable in a non-invasive screening programme; the Office-Based, BMI-based Framingham equation is, to our knowledge, the only laboratory-free option. The cardiovascular construct is therefore used for relative, within-cohort risk ordering and hypothesis generation, not as a calibrated individual prognosis.

Construct circularity: The MUO score and the patient similarity network were built in part from VFL, BMI and age; correlations and community structure involving these constructs therefore reflect internal construct consistency and require independent, imaging-based and longitudinal validation.

Conservative, non-invasive FINDRISC scoring: Because laboratory glucose values and systematically recorded family history were unavailable in this non-invasive screening programme, the corresponding FINDRISC components were scored as zero. This deliberately conservative approach underestimates the true Finnish Diabetes Risk Score and produces a low apparent prevalence of elevated risk (0.6%); the score is consequently a statistical risk-stratification construct, not a diagnostic estimate. The trade-off is intentional and appropriate for screening: scoring missing components as zero maximises specificity and minimises false-positive labelling, so that the small subgroup nonetheless flagged as elevated is highly likely to be genuinely at risk. As no more accurate laboratory-free alternative exists, the modified FINDRISC is retained here for relative risk ordering and hypothesis generation rather than individual diagnosis.

No comparison against laboratory or imaging reference standards: the framework was evaluated for internal consistency only. No fasting glucose, HbA1c, lipid profile, insulin-resistance index, inflammatory marker or CT/MRI-based visceral fat measurement was available in this screening programme. It is therefore not possible to determine whether the constructs described here agree with, or add anything to, established laboratory-based risk assessment. In particular, we cannot state whether the BIA-derived MUO score identifies the same individuals as a laboratory definition of Metabolically Unhealthy Obesity, nor whether the framework improves risk prediction beyond existing clinical methods. Demonstrating incremental value would require a cohort in which the non-invasive framework and a full laboratory and imaging panel are obtained in the same participants; until then, the framework should be regarded as a hypothesis-generating screening construct rather than a clinical instrument.

Uncertain accuracy of the BIA-defined TOFI-like definition: the combination of BMI < 25 kg/m^2^ and VFL ≥ 10 forms a pragmatic bioimpedance screen, but bioimpedance estimates of visceral adiposity are least accurate precisely in normal-weight individuals, in whom the absolute visceral fat area is small and the impedance signal is dominated by the subcutaneous and lean compartments [53,54]. Without imaging confirmation, the true prevalence of visceral adiposity in this subgroup may be either over- or underestimated. The reported 5.2% should therefore be read as the prevalence of a BIA-defined operational phenotype, not of imaging-confirmed TOFI.

Analyst-specified network parameters: the feature weights, the target network density and the Louvain resolution were fixed a priori rather than optimised, and different choices would yield somewhat different partitions. The benchmark in Section 3.7.1 partly addresses this by showing convergence with unweighted conventional clustering, but a formal sensitivity analysis across weighting schemes remains to be performed.

### 4.7. Clinical Implications

The identification of 21.8% high-MUO and 5.2% BIA-defined TOFI-like phenotypes in a young, ostensibly healthy population is hypothesis-generating. The points below are directions for future evaluation, not recommendations for practice: this study is cross-sectional, contains no follow-up and no outcome events, and therefore cannot support any claim of clinical benefit.

Screening Strategy: whether DSM-BIA measurement adds usable information to standard BMI assessment in primary care is a question for prospective studies that include laboratory and, ideally, imaging comparators.

Resource Allocation: network-based stratification generates candidate priority groups (for example the older, high-cardiovascular-risk community C6) whose usefulness for targeting early lifestyle intervention would need to be tested in a trial or longitudinal cohort.

Early Prevention: earlier recognition of adverse body-composition phenotypes might, if prognostic relevance were established longitudinally, create an opportunity for preventive intervention during a modifiable period [56].

These implications are exploratory; none of the constructs studied here have been validated against clinical endpoints in this cohort, and all require longitudinal validation before clinical adoption.

### 4.8. Future Directions

Prospective cohort follow-up to validate predictive accuracy and replace pseudo-labels with true outcome data;

Multi-centre validation across diverse populations;

Integration of additional biomarkers (lipid panel, HbA1c) for comparison;

Mobile application development for point-of-care use;

Cost-effectiveness analysis of DSM-BIA screening;

Longitudinal two-timepoint analysis using the built-in two-dataset comparison pipeline;

External validation of the network-derived (Louvain) phenotypic communities against clinical outcomes;

Head-to-head comparison with laboratory-based risk assessment (fasting glucose, HbA1c, lipid profile, insulin-resistance indices, inflammatory markers) and with imaging-based visceral fat quantification, to establish whether the non-invasive framework carries incremental information;

Systematic sensitivity analysis of the network parameters (feature weights, target density, resolution) and comparison with additional graph-based and probabilistic clustering methods;

Adaptation and prospective testing of the pipeline in ageing research, where DSM-BIA-derived visceral fat level, skeletal muscle mass and percent body fat are candidate components of biological-age models.

## 5. Conclusions

In this large young-adult screening cohort, a non-invasive methodological framework that integrates bioelectrical impedance-derived body-composition phenotyping, three complementary non-invasive risk constructs and patient similarity network stratification behaved consistently with their predefined design assumptions: all six predefined hypotheses (H1–H6) were satisfied (Table 11). The framework surfaced body-composition heterogeneity—most notably a BIA-defined TOFI-like phenotype—that BMI alone does not capture, and it did so with high internal feature-space separability and statistically robust network structure. Benchmarking against conventional clustering showed that the same phenotypic axes are recovered by independent method families, while the network additionally determines the number of subgroups from the data and permits a null-model test of the partition.

The present study should be interpreted as a methodological and hypothesis-generating investigation, not as a validation of a clinically deployable risk-prediction tool. The framework demonstrated internal construct consistency and phenotypic separability, and BMI alone did not capture all BIA-defined adiposity patterns observed in this cohort. Because no laboratory, imaging or outcome data were available, no statement about diagnostic validity, prognostic accuracy or clinical utility can be made. Prospective, externally validated studies incorporating laboratory biomarkers, imaging-based visceral fat assessment and longitudinal cardiometabolic outcomes will be required before such claims can be considered.

## Data Availability

The data presented in this study are available on request from the corresponding author. The data are not publicly available due to privacy restrictions in accordance with GDPR.

## Supplementary Materials

The following supporting information can be downloaded at: https://www.mdpi.com/article/10.3390/life16081336/s1, Supplementary Methods S1: Alternative similarity kernels, the modularity function and the adaptive threshold derivation. Table S1: Office-Based Framingham risk strata. Table S2: Scoring components of the modified, proxy-based FINDRISC. Table S3: Risk categories of the original FINDRISC—for reference only. Table S4: Proxy variables used for the unavailable FINDRISC components. Table S5: Complete pairwise correlation matrix. Table S6: Patient similarity network versus conventional clustering and dimensionality-reduction approaches.

## Abbreviations

The following abbreviations are used in this manuscript:

ACE	American Council on Exercise
ANOVA	Analysis of Variance
ARI	Adjusted Rand Index
AUC	Area Under the Curve
BIA	Bioelectrical Impedance Analysis
BMI	Body Mass Index
BMR	Basal Metabolic Rate
BP	Blood Pressure
CCC	Concordance Correlation Coefficient
CT	Computed Tomography
CVD	Cardiovascular Disease
DBP	Diastolic Blood Pressure
DSM-BIA	Direct Segmental Multi-Frequency Bioelectrical Impedance Analysis
DXA	Dual-Energy X-ray Absorptiometry
ESC	European Society of Cardiology
EWGSOP2	European Working Group on Sarcopenia in Older People 2
FDR	False Discovery Rate
FINDRISC	Finnish Diabetes Risk Score
GDPR	General Data Protection Regulation
HbA1c	Glycated Haemoglobin
HDBSCAN	Hierarchical Density-Based Spatial Clustering of Applications with Noise
HDL	High-Density Lipoprotein
HÖOK	National Union of Students in Hungary (Hallgatói Önkormányzatok Országos Konferenciája)
HT	Hypertension
IDF	International Diabetes Federation
IPAQ	International Physical Activity Questionnaire
IQR	Interquartile Range
MetS	Metabolic Syndrome
MRI	Magnetic Resonance Imaging
MUO	Metabolically Unhealthy Obesity
NMI	Normalised Mutual Information
PBF	Percent Body Fat
PCA	Principal Component Analysis
PSN	Patient Similarity Network
RBF	Radial Basis Function
ROC	Receiver Operating Characteristic
SBP	Systolic Blood Pressure
SD	Standard Deviation
SMM	Skeletal Muscle Mass
T2DM	Type 2 Diabetes Mellitus
TOFI	Thin Outside, Fat Inside
UMAP	Uniform Manifold Approximation and Projection
VFL	Visceral Fat Level
