# Supplementary material for: A Methodological Framework for Non-Invasive Body-Composition Phenotyping in Young Adults: Integrating Bioelectrical Impedance and Patient Similarity Networks
Source: Life (Basel). 2026 Aug 14;16(8):1336. doi: 10.3390/life16081336 (PMC13514111; doi:10.3390/life16081336)
Supplement: Supplementary file 1 [file life-16-01336-s001.zip › life-4444366-supplementary.pdf]

Supplementary Materials

A Methodological Framework for Non-Invasive Body-Composition Phenotyping in Young Adults: Integrating Bioelectrical Impedance and Patient Similarity Networks

Róbert László Nagy, Bence Bombera, Csongor István Szepesi, Nóra Horváth, Viktor Rekenyi, László Róbert Kolozsvári

Supplementary Methods S1. Similarity, thresholding and community detection

S1.1 Feature vector and weighting

Each participant i is represented by the nine-dimensional feature vector x_i = (age, BMI, SMM, VFL, PBF, SBP, CVD risk, FINDRISC, MUO), standardised feature-wise to zero mean and unit variance and then scaled by the a priori clinical weight vector w = (1.5, 2.0, 1.5, 2.0, 1.5, 1.5, 2.5, 2.0, 1.8):

$$z_{ij} = (x_{ij} - \mu_j) / \sigma_j , \quad \tilde{x}_i = w \odot z_i$$

where $\odot$ denotes element-wise multiplication. All n = 1,662 participants with a complete feature vector were included.

S1.2 Primary similarity: weighted cosine

$$S(i, j) = \langle w \odot z_i , w \odot z_j \rangle / (\|w \odot z_i\|_2 \cdot \|w \odot z_j\|_2)$$

with $\|\cdot\|_2$ the Euclidean norm. Every result reported in the manuscript uses this kernel.

S1.3 Alternative similarity kernels (implemented but not used in the reported results)

Weighted Euclidean distance, inverted to a similarity:

$$S_{euc}(i, j) = 1 / (1 + \| w \odot (z_i - z_j) \|_2)$$

Gaussian (radial basis function) kernel:

$$S_{rbf}(i, j) = \exp(- \| w \odot (z_i - z_j) \|_2^2 / (2 \sigma^2)), \quad \sigma = 1.0 \text{ by default}$$

S1.4 Adaptive threshold selection

Rather than fixing an arbitrary similarity cut-off, the target edge density scales inversely with cohort size so that the average degree remains interpretable (approximately 75 neighbours):

$$\rho_{target}(n) = \mathrm{clip}(125 / n , 0.02 , 0.30)$$

The similarity threshold τ is the corresponding upper quantile of the pairwise similarity distribution:

$$\tau = \mathrm{clip}(\mathrm{Quantile}_{1 - \rho_{target}} (\{ S(i, j) : i < j \}) , 0.50 , 0.95)$$

For the present cohort (n = 1,662) the formula yields ϱ_target ≈ 0.045; the realised network applied a target density of 0.05, giving τ = 0.832, 69,015 edges and an average node degree of 83.05. An excessively dense graph renders all participants mutually similar and is uninformative; an excessively sparse graph fragments into isolated nodes with no community structure.

S1.5 Edge creation and modularity

An unweighted edge is created whenever the similarity exceeds the threshold, A_ij = 1 if S(i, j) ≥ τ and 0 otherwise. Community detection uses the Louvain algorithm with a deterministic seed (42) and resolution γ = 1.0, maximising

$$Q = (1 / 2m)\ \Sigma\_ij\ [\ A\_ij - \gamma \cdot (k\_i\ k\_j / 2m)\ ] \cdot \delta(c\_i, c\_j)$$

where A_ij is the binary adjacency matrix, k_i the degree of node i, m the total number of edges, c_i the community assignment of node i, and δ(·,·) the Kronecker delta. Vertices of degree 0 cannot be assigned to a community and are excluded from the partition (five participants).

S1.6 Configuration-model permutation test

The observed modularity is compared with a degree-preserving null distribution generated by repeated double-edge-swap randomisation of the observed graph, which preserves the exact degree sequence. Louvain community detection is re-run on each of k = 200 null replicates and its modularity recorded; the empirical one-sided p-value is

$$p = (\ 1 + \#\{\ Q\_null \geq Q\_obs\ \}\ )\ /\ (\ 1 + k\ )$$

Observed Q = 0.6296 versus a null of 0.0815 ± 0.0015 (mean ± SD), p = 0.005.

S1.7 Benchmarking procedure (Table S6)

All comparators were applied to the identical weighted, standardised nine-feature matrix. Methods requiring the number of clusters to be fixed in advance were matched to the nine communities recovered by Louvain. Principal component analysis retained the three components explaining ≥ 80% of the variance. UMAP used n_neighbors = 15, min_dist = 0.1 and two components; HDBSCAN used a minimum cluster size of 25. Internal validity (silhouette, Calinski–Harabasz, Davies–Bouldin) was computed in the original weighted feature space on assigned points only, so that all methods are scored on the same space. Agreement with the Louvain partition is reported as the adjusted Rand index (ARI) and normalised mutual information (NMI). Reproducibility is the mean pairwise ARI over 25 independent 80% subsamples, restricted to the intersection of each pair of subsamples. Subgroup recovery is the best single-cluster F1 score against two reference subgroups: the BIA-defined TOFI-like phenotype (BMI < 25 kg/m^2 and VFL ≥ 10; n = 83 within the networked cohort) and older participants at elevated cardiovascular risk (age ≥ 40 years and Framingham ordering ≥ 5%). The full script is provided as clustering_benchmark.py.

Table S1. Office-Based Framingham risk strata

Strata follow the 2021 European Society of Cardiology guidelines on cardiovascular disease prevention in clinical practice. In the present cohort these labels denote strata of a relative, within-cohort ordering and not calibrated absolute 10-year probabilities, because the equation was applied off-label below 30 years of age.

10-year risk (%)	Stratum label
< 5	Low
5–10	Moderate
10–20	High
≥ 20	Very high

Table S2. Scoring components of the modified, proxy-based FINDRISC

Components marked "not available" were scored conservatively as zero for every participant; the resulting instrument is therefore not the validated FINDRISC.

Factor	Criteria	Points	Availability in this study
Age (years)	< 45 / 45–54 / 55–64 / ≥ 65	0 / 2 / 3 / 4	Available
BMI (kg/m²)	< 25 / 25–30 / > 30	0 / 1 / 3	Available
Waist circumference (cm), M/F	< 94/80 / 94–102/80–88 / > 102/88	0 / 3 / 4	Measured in 521; otherwise estimated from BMI and sex
Physical activity	≥ 30 min/day / < 30 min/day	0 / 2	Available (IPAQ)
Daily vegetables	Yes / No	0 / 1	Not available — scored 0
Antihypertensive medication	No / Yes	0 / 2	Proxy: self-reported hypertension or antihypertensive medication
History of high blood glucose	No / Yes	0 / 5	Not available — scored 0
Family history of diabetes	None / 2nd degree / 1st degree	0 / 3 / 5	Not available — scored 0

Table S3. Risk categories of the ORIGINAL FINDRISC—for reference only

These categories and the associated 10-year risks are calibrated for the complete, validated instrument. They are NOT applicable to the modified proxy-based score used in this study, in which several components were systematically scored as zero, and they are not used to interpret any result reported in the manuscript.

Score	Category (original instrument)	10-year T2DM risk (original instrument)
< 7	Very low	~1%
7–11	Low	~4%
12–14	Moderate	~17%
15–20	High	~33%
> 20	Very high	~50%

Table S4. Proxy variables used for the unavailable FINDRISC components

Original component	Proxy / handling	Observed prevalence	Rationale
Antihypertensive medication history	Self-reported chronic condition ("hypertension") OR antihypertensive medication	2.6% (44/1,684)	Consistent with young-adult treatment rates
History of high blood glucose	Not available — scored 0	n/a	Absence of data treated as negative to avoid overestimation

Family history of diabetes (1st degree)	Not recorded — scored 0	0/1,684	Not systematically captured in this screening dataset
Family history of hypertension	Not recorded — scored 0	0/1,684	Not systematically captured in this screening dataset
Daily vegetable consumption	Not recorded — scored 0	n/a	Dietary data were not captured; scored conservatively as 0

Table S5. Complete pairwise correlation matrix

All 45 pairwise associations among the 10 continuous variables available in this screening dataset, sorted by descending |r|. Pearson r with 95% Fisher-z confidence intervals and Spearman ρ are reported; p-values are adjusted for multiple comparisons with the Benjamini–Hochberg false discovery rate procedure across all pairs. The final column states whether the two variables share components by construction; associations so flagged quantify construct consistency rather than independent biological relationships (Section 2.6 of the main text). Abbreviations: BMI, body mass index; VFL, visceral fat level; PBF, percent body fat; SMM, skeletal muscle mass; SBP/DBP, systolic/diastolic blood pressure; CVD, cardiovascular disease; FINDRISC, modified Finnish Diabetes Risk Score; MUO, metabolically unhealthy obesity.

**Variable pair**	**n**	**Pearson r**	**95% CI**	**q (BH)**	**Spearman ρ**	**q_ρ (BH)**	**Shared components**
VFL–PBF	1,684	0.855	[0.84, 0.87]	< 0.001	0.847	< 0.001	independent measurements
VFL–MUO	1,684	0.846	[0.83, 0.86]	< 0.001	0.840	< 0.001	shares components with MUO
PBF–MUO	1,684	0.743	[0.72, 0.76]	< 0.001	0.669	< 0.001	shares components with MUO
BMI–VFL	1,684	0.728	[0.70, 0.75]	< 0.001	0.676	< 0.001	independent measurements
Age–CVD risk	1,662	0.666	[0.64, 0.69]	< 0.001	0.072	0.004	shares components with Framingham
BMI–MUO	1,684	0.637	[0.61, 0.66]	< 0.001	0.661	< 0.001	shares components with MUO
SMM–SBP	1,662	0.589	[0.56, 0.62]	< 0.001	0.599	< 0.001	independent measurements
VFL–FINDRISC	1,684	0.558	[0.52, 0.59]	< 0.001	0.547	< 0.001	independent measurements
BMI–SMM	1,684	0.549	[0.51, 0.58]	< 0.001	0.574	< 0.001	independent measurements
SBP–DBP	1,662	0.507	[0.47, 0.54]	< 0.001	0.524	< 0.001	independent measurements
PBF–FINDRISC	1,684	0.504	[0.47, 0.54]	< 0.001	0.521	< 0.001	independent measurements
BMI–FINDRISC	1,684	0.490	[0.45, 0.53]	< 0.001	0.407	< 0.001	shares components with FINDRISC
PBF–SMM	1,684	-0.470	[-0.51, -0.43]	< 0.001	-0.501	< 0.001	independent measurements
FINDRISC–MUO	1,684	0.457	[0.42, 0.49]	< 0.001	0.428	< 0.001	both composite
BMI–SBP	1,662	0.444	[0.40, 0.48]	< 0.001	0.449	< 0.001	independent measurements
BMI–PBF	1,684	0.396	[0.35, 0.44]	< 0.001	0.317	< 0.001	independent measurements
SBP–CVD risk	1,662	0.361	[0.32, 0.40]	< 0.001	0.552	< 0.001	shares components with Framingham
CVD risk–FINDRISC	1,662	0.359	[0.32, 0.40]	< 0.001	0.185	< 0.001	both composite
Age–FINDRISC	1,684	0.326	[0.28, 0.37]	< 0.001	0.021	0.390	shares components with FINDRISC
BMI–CVD risk	1,662	0.322	[0.28, 0.36]	< 0.001	0.430	< 0.001	shares components with Framingham
Age–BMI	1,684	0.320	[0.28, 0.36]	< 0.001	0.346	< 0.001	independent measurements
DBP–MUO	1,663	0.303	[0.26, 0.35]	< 0.001	0.336	< 0.001	independent measurements
Age–VFL	1,684	0.286	[0.24, 0.33]	< 0.001	0.263	< 0.001	independent measurements
SBP–MUO	1,662	0.286	[0.24, 0.33]	< 0.001	0.377	< 0.001	shares components with MUO
BMI–DBP	1,663	0.277	[0.23, 0.32]	< 0.001	0.255	< 0.001	independent

							measurements
SMM–CVD risk	1,662	0.269	[0.22, 0.31]	< 0.001	0.501	< 0.001	independent measurements
Age–MUO	1,684	0.248	[0.20, 0.29]	< 0.001	0.240	< 0.001	independent measurements
VFL–DBP	1,663	0.239	[0.19, 0.28]	< 0.001	0.228	< 0.001	independent measurements
CVD risk–MUO	1,662	0.233	[0.19, 0.28]	< 0.001	0.268	< 0.001	both composite
DBP–CVD risk	1,662	0.231	[0.18, 0.28]	< 0.001	0.278	< 0.001	independent measurements
VFL–CVD risk	1,662	0.200	[0.15, 0.25]	< 0.001	0.121	< 0.001	independent measurements
Age–PBF	1,684	0.192	[0.15, 0.24]	< 0.001	0.126	< 0.001	independent measurements
Age–DBP	1,663	0.167	[0.12, 0.21]	< 0.001	0.197	< 0.001	independent measurements
PBF–SBP	1,662	-0.166	[-0.21, -0.12]	< 0.001	-0.205	< 0.001	independent measurements
SMM–DBP	1,663	0.152	[0.10, 0.20]	< 0.001	0.159	< 0.001	independent measurements
PBF–DBP	1,663	0.140	[0.09, 0.19]	< 0.001	0.129	< 0.001	independent measurements
DBP–FINDRISC	1,663	0.129	[0.08, 0.18]	< 0.001	0.114	< 0.001	independent measurements
VFL–SBP	1,662	0.126	[0.08, 0.17]	< 0.001	0.096	< 0.001	independent measurements
Age–SBP	1,662	0.118	[0.07, 0.17]	< 0.001	0.147	< 0.001	independent measurements
Age–SMM	1,684	0.114	[0.07, 0.16]	< 0.001	0.220	< 0.001	independent measurements
SBP–FINDRISC	1,662	0.110	[0.06, 0.16]	< 0.001	0.053	0.033	independent measurements
SMM–MUO	1,684	0.040	[-0.01, 0.09]	0.112	0.100	< 0.001	shares components with MUO
PBF–CVD risk	1,662	0.032	[-0.02, 0.08]	0.206	-0.145	< 0.001	independent measurements
SMM–FINDRISC	1,684	-0.015	[-0.06, 0.03]	0.545	-0.068	0.006	independent measurements
VFL–SMM	1,684	-0.004	[-0.05, 0.04]	0.879	-0.031	0.213	independent measurements

Table S6. Patient similarity network versus conventional clustering and dimensionality-reduction approaches

All methods were applied to the identical weighted, standardised nine-feature matrix (n = 1,662). Silhouette, Calinski–Harabasz (CH) and Davies–Bouldin (DB) indices are computed in that feature space on assigned points only; higher silhouette and CH, and lower DB, indicate more compact clusters. ARI and NMI quantify agreement with the Louvain reference partition. Reproducibility is the mean pairwise ARI over 25 independent 80% subsamples. Subgroup recovery is the best single-cluster F1 against the BIA-defined TOFI-like phenotype and against older participants at elevated cardiovascular risk. Note that centroid-based methods optimise the same Euclidean compactness criterion that the silhouette and CH indices measure, so their advantage on those columns is expected and is not evidence of a better phenotypic partition. Applying the conventional silhouette criterion to select k for k-means returns k = 2 (silhouette 0.374; at k = 9, 0.216), which merges the small clinically relevant subgroups. Source: clustering_benchmark.py; raw output in clustering_benchmark_results.txt.

**Method**	**k**	**k determined**	**Un-assigned**	**Silh.**	**CH**	**DB**	**ARI vs Louvain**	**NMI vs Louvain**	**Repro-ducibility**	**TOFI-like F1**	**Older high-CVD F1**
PSN + Louvain (proposed)	9	data-driven	5	0.054	239	1.35	1.00	1.00	0.73	0.29	0.70
k-means	9	pre-specified	0	0.216	496	1.32	0.50	0.63	0.77	0.60	0.54
Ward hierarchical	9	pre-specified	0	0.180	427	1.43	0.42	0.60	0.56	0.54	0.57
PCA (3 PCs, ≥80% var.) + k-means	9	pre-specified	0	0.193	479	1.42	0.40	0.57	0.83	0.57	0.62
UMAP (2D) + HDBSCAN	4	data-driven	24	−0.129	49	1.71	0.12	0.33	0.61	0.12	0.09
